# Current advances in the structural biology and molecular engineering of PETase

**DOI:** 10.3389/fbioe.2023.1263996

**Published:** 2023-09-19

**Authors:** Fei Liu, Tao Wang, Wentao Yang, Yingkang Zhang, Yuming Gong, Xinxin Fan, Guocheng Wang, Zhenhua Lu, Jianmin Wang

**Affiliations:** ^1^ School of Biological Science, Jining Medical University, Rizhao, China; ^2^ College of Chemical and Biological Engineering, Zhejiang University, Hangzhou, Zhejiang, China; ^3^ School of Pharmacy, Jining Medical University, Rizhao, China

**Keywords:** Poly(ethylene terephthalate), biodegradation, *Is*PETase, MHETase, protein engineering

## Abstract

Poly(ethylene terephthalate) (PET) is a highly useful synthetic polyester plastic that is widely used in daily life. However, the increase in postconsumer PET as plastic waste that is recalcitrant to biodegradation in landfills and the natural environment has raised worldwide concern. Currently, traditional PET recycling processes with thermomechanical or chemical methods also result in the deterioration of the mechanical properties of PET. Therefore, it is urgent to develop more efficient and green strategies to address this problem. Recently, a novel mesophilic PET-degrading enzyme (*Is*PETase) from *Ideonella sakaiensis* was found to streamline PET biodegradation at 30°C, albeit with a lower PET-degrading activity than chitinase or chitinase-like PET-degrading enzymes. Consequently, the molecular engineering of more efficient PETases is still required for further industrial applications. This review details current knowledge on *Is*PETase, MHETase, and *Is*PETase-like hydrolases, including the structures, ligand‒protein interactions, and rational protein engineering for improved PET-degrading performance. In particular, applications of the engineered catalysts are highlighted, including metabolic engineering of the cell factories, enzyme immobilization or cell surface display. The information is expected to provide novel insights for the biodegradation of complex polymers.

## 1 Introduction

Plastics are highly useful synthetic materials and nearly omnipresent in our daily life, which have also revolutionized modern life in various sectors, such as clothing, automotive, medical, and electronic industries. Among them, polyethylene terephthalate (PET) is the most widely used synthetic polyester plastic, especially in the packaging industry. However, with the increasing consumption, a remarkable part of plastics is accumulated in landfills and the natural environment as plastic waste (García-Martínez and Collar, 2023; Kwon, 2023). Only 9% of all plastics ever produced were estimated to be recycled and 12% were incinerated (Tiso et al., 2021). Caused by the strong physical durability and chemical stability, PET is usually recalcitrant to biodegradation. As a result, the white crisis has raised a globally burgeoning concern (Sohn et al., 2020).

To deal with the increasingly serious problem of plastic waste, on the one hand researchers have been searching for alternatives to petroleum-derived plastics to replace the conventional plastics. Bioplastics (Venâncio, Lopes, and Oliveira, 2022; Jayakumar et al., 2023), as the novel biodegradable materials made of renewable sources (e.g., plants) or biological material (e.g., starch), have drawn great attentions globally. They are usually renewable, biodegradable, compostable, and environment friendly, such as polyhydroxyalkanoates (PHAs) (Khatami et al., 2021), Mater-bi^®^(Magni et al., 2020). Definition of bioplastic is not consensual, however based on the sources, bioplastics could be classified into: the bio-based and biodegradable polymers; the bio-based but bio-nondegradable, and the biodegradable polymers synthesized chemically (Arif et al., 2023). Though (compostable) bioplastics still represent less than 0.5% of global plastics production (Bioplastics, 2023), the global demand for the bioplastics is growing rapidly. Even so, there are still many challenges to deal with associated with the bioplastics (Dolci, Rigamonti, and Grosso, 2023). Firstly, efficient degradation of bioplastics usually combined with the traditional plastic are often time-consuming, laborious, and costly. In addition, limited and slow degradability of different bioplastics would be caused by different biological processes such as the aerobic or anaerobic conditions and thermophilic or mesophilic treatment. The main purpose of this review is to provide an overview for efficient management of petroleum-based plastics waste (especially PET material) catalysed by the PETase especially the *Is*PETase or the mutants. Therefore, details on the bioplastics were not discussed, which could be found in other excellent studies (Ali et al., 2022; Ghasemlou et al., 2022; Jayakumar et al., 2023; Park et al., 2023; Pascoe Ortiz, 2023; Rajeshkumar et al., 2023).

On the other hand, recently the concept of circular carbon economy based on biotechnological plastic recycling has become a thriving research area in recent years (Ru, Huo, and Yang, 2020; Ellis et al., 2021a; Kakadellis and Rosetto, 2021; Simon et al., 2021). In addition to the traditional mechanical recycling and chemical recycling (Mederake, 2022), biocatalytic depolymerization catalyzed by PET-degrading enzymes (e.g., PETase) has emerged as a promising and sustainable alternative for PET upcycling and enable circularity (Magalhães, Cunha, and Sousa, 2021; Cao et al., 2022; Lai et al., 2022; Ambrose-Dempster et al., 2023). A variety of naturally occurring plastic degrading enzymes have been discovered and characterized from microbial sources (Kawai, 2021; Erickson et al., 2022; Khairul Anuar et al., 2022; Wei et al., 2022; Zhu, Wang, and Wei, 2022). These PET-hydrolysing enzymes breaking the PET polymer into diverse water-soluble products, such as terephthalic acid (TPA), ethylene glycol (EG), and (mono-(2-hydroxyehyl)terephthalic acid (MHET). Generally, PET-degrading enzymes can be classified into thermophilic enzymes and mesophilic enzymes according to the optimal reaction temperature. In 2005, a hydrolase was identified that could hydrolyse two kinds of PET films at 55°C and is believed to be the first reported PET-degrading enzyme (RJ et al., 2005).

To date, around 89 different enzymes (PAZy database (Buchholz et al., 2022), https://pazy.eu/doku.php) with PET-degrading ability have been discovered and characterized (Magalhães, Cunha, and Sousa, 2021), and most of them can be classified in the subfamily of carboxylic ester hydrolases (Table 1, EC 3.1.1.x), such as carboxylesterase (EC 3.1.1.1) (von Haugwitz et al., 2022), lipases (Moyses et al., 2021), cutinases (EC 3.1.1.74) (Wei and Zimmermann, 2017), and arylesterase (EC 3.1.1.2) (Austin et al., 2018). Great progress has been made involving cutinases or cutinase-like PET-degrading enzymes (Sulaiman et al., 2012; Xue et al., 2021; Brandenberg, Schubert, and Kruglyak, 2022; Jia et al., 2022; Lu et al., 2022), and there are several excellent reviews of cutinases relevant to PET hydrolysis (Zumstein et al., 2017; Tamoor et al., 2021; Urbanek, Kosiorowska, and Mirończuk, 2021; Kumar et al., 2022).

**TABLE 1:** Typical PET-degrading enzymes.

PET degrading enzymes	Subfamily	Protein and variants		Ref
Carboxylic ester hydrolases	Carboxylesterase	TfCa from *T. fusca* KW3		(Billig et al., 2010)
	Lipases	Lipases from *Aspergillus oryzae* CCUG 33812*, Candida antarctica* (CALB) and *Pichia pastoris*		Wang et al., 2008; Eberl et al., 2009; Carniel et al., 2017; Gao et al., 2017; Świderek et al., 2023
	Esterase	Esterase from *Thermobifida halotolerans* (Thh_Est)		(Doris Ribitsch et al., 2012)
	*p*-nitrobenzylesterase	*p*-*nitrobenzylesterase from Bacillus subtilis* (BsEstB)		(D. Ribitsch et al., 2011)
	Cutinases	Cut190	Cut190 ^(L136F/Q138A/S226P/R228S/D250CE296C/Q123H/N202H/K305del/L306del/N307del)^	(Kawai et al., 2022)
		LCC	LCC^ICCG^ (LCC^F243I/D238C/S283C/Y127G^)	(Tournier et al., 2020)
		*Tf*Cut2	*Tf*Cut2^G62A/F249R^, *Tf*Cut2 ^S121P/D174S/D204P^	(Li et al., 2022; Mrigwani et al., 2023)
	Cutinase-like enzyme	*Is*PETase	Dura (*Is*PETase^S214H−I168R−W159H−S188Q−R280A−A180I−G165A−Q119Y−L117F−T140D^)	(Y. Cui, Chen, et al., 2021b)
			Fast-PETase (*Is*PETase^S121E/D186H/R224Q/N233K/R280A^)	(Lu et al., 2022)
			TS-PETase (*Is*PETase^R280A S121E D186H N233C S282C^)	(Zhong-Johnson, Voigt, and Sinskey, 2021)
			HotPETase (*Is*PETase^S121E/D186H/R280A/P181V/S207R/S214Y/Q119K/S213E/N233C/S282C/R90T/Q182M/N212K/R224L/S58A/S61V/K95N/M154G/N241C/K252M/T270Q^)	(Bell, Smithson, and Kilbride, 2022)
			ThermoPETase (*Is*PETase^S121E/D186H/R280A^)	(Joo et al., 2018)
		BhrPETase	BhrPETase^H184S/F93G/F209I/S213K^	(X.Q. Chen, Guo, et al., 2022b)
		BbPETase	BbPETase^S335N/T338I/M363I/N365G^	(C.C. Chen et al., 2021)
		*Is*MHETase	MHETase^R411K/S416A/F424I^	(Hye Young Sagong et al., 2020)

In 2016, two novel enzymes [ISF6_4831 (*Is*PETase) and ISF6_0224 (*Is*MHETase)] were identified and determined to be capable of hydrolysing low-crystallinity (1.9%) PET film from *Ideonella sakaiensis* 201-F6 at 30°C (Yoshida et al., 2016). *Is*PETase shows great potential for industrially relevant PET degradation because of its relatively high degradation activities and low catalytic temperatures (Maity et al., 2021). The increasing numbers of known *Is*PETase variants (Table 2), such as DuraPETase (Y. Cui, Chen, et al., 2021b), Fast-PETase (Lu et al., 2022) and TS-PETase (Zhong-Johnson, Voigt, and Sinskey, 2021), have further increased their application potential. In particular, the engineered thermostable PETase mutant (HotPETase) shows more efficient cry-PET hydrolysing activity at 40°C than LCC^ICCG^ at 65°C (Bell, Smithson, and Kilbride, 2022). All these advantages highlight the promise of enzymatic industrial PET depolymerization with minimal additional pretreatment.

**TABLE 2:** The catalytic activities of the above-mentioned enzymes with promising potentials are summarized.

Enzyme	Mutants (or wild type)	Catalytic activities	Optimum temperature (°C)	Thermostability (T_m_, °C)	Ref
*Is*PETase	*Is*PETase	0.3 mM products were released for 18 h at 30°C against the PET film (diameter, 6 mm)	40	46.8	
	DuraPETase (*Is*PETase^S214H−I168R−W159H−S188Q−R280A−A180I−G165A−Q119Y−L117F−T140D^)	3.1 mM products were released for 10 days at 30°C, against the PET films (crystallinity, 30%)	37	77	(Y. Cui, Chen, et al., 2021b)
	Fast-PETase (*Is*PETase^S121E/D186H/R224Q/N233K/R280A^)	33.8 mM products were released in 96 h against the gf-PET film (6 mm in diameter, roughly 11.4 mg)	50	—	(Lu et al., 2022)
	TS-PETase (*Is*PETase^R280A/S121E/D186H/N233C/S282C^)	∼4 mM products were released for 6 days at 30°C, against the ¼“-diameter amorphous circular PET pellets	58	69.4 ± 0.3	(Zhong-Johnson, Voigt, and Sinskey, 2021)
	HotPETase (*Is*PETase^S121E/D186H/R280A/P181V/S207R/S214Y/Q119K/S213E/N233C/S282C/R90T/Q182M/N212K/R224L/S58A/S61V/K95N/M154G/N241C/K252M/T270Q^)	6.07 mM soluble monomer within 5 h at 60°C with a degree of depolymerization of 31%, against the cryPET (0.4% crystallinity, 4 g/L)	60	82.5	(Bell, Smithson, and Kilbride, 2022)
	ThermoPETase (*Is*PETase^S121E/D186H/R280A^)	0.12 mM soluble monomer within 72 h at 40°C against a PET film	40	57.62	(Son et al., 2019)
BhrPETase	BhrPETase^H184S/F93G/F209I/S213K^	The degradation rate against the plastic bottles reached to 73% at 60°C, 50 mg_enzyme_g_PET_^-1^ in 96 h	60	—	(X.Q. Chen, Guo, et al., 2022a)
BbPETase	BbPETase^S335N/T338I/M363I/N365G^	∼2 mM soluble monomer within 3 days at 40°C against a mcPET (crystallinity 17%)	40	63	(H. Y. Sagong et al., 2022)
*Is*MHETase	MHETase^R411K/S416A/F424I^	∼8 µM soluble monomer within 72 h at 30°C against the PETase^S121A/D186H/R280A^-treated PET film	—	—	(Hye Young Sagong et al., 2020)

(/:not provided).

This review presents a brief overview of the recent advances in *Is*PETase, *Is*MHETase and special cutinases with PET-degrading activities, including their structures, catalytic mechanisms, and applications. In particular, rational protein engineering based on macromolecular structures is highlighted. Finally, further perspectives on the industrial applications of enzyme-catalysed PET degradation are discussed.

## 2 Promising mesophilic enzymes for PET degradation

### 2.1 PET hydrolase from *Ideonella sakaiensis*

#### 2.1.1 *Is*PETase

In 2016, a PET-degrading bacterium, *Ideonella sakaiensis* 201-F6, was identified and found to grow on low-crystallinity (1.9%) PET film at 30°C, degrading the PET film almost completely after 6 weeks (Yoshida et al., 2016). To more PET-degrading enzymes (ISF6_4831 and ISF6_0224) from *I. sakaiensis* 201-F6 were determined to be capable of degrading PET into MHET (ISF6_4831, also named *Is*PETase) and MHET into TPA (ISF6_0224, also named MHETase). In addition, *Is*PETase is currently known as the most efficient PET-degrading cutinase operating at room temperature (Kan et al., 2021), although it also suffers from the common problem of low thermostability at the glass transition temperature (*T*_g_) of PET. Crystal structures of *Is*PETase have been reported to illustrate the structure-activity relationship, catalytic mechanism, and rational site-directed mutagenesis.

**FIGURE 1:**
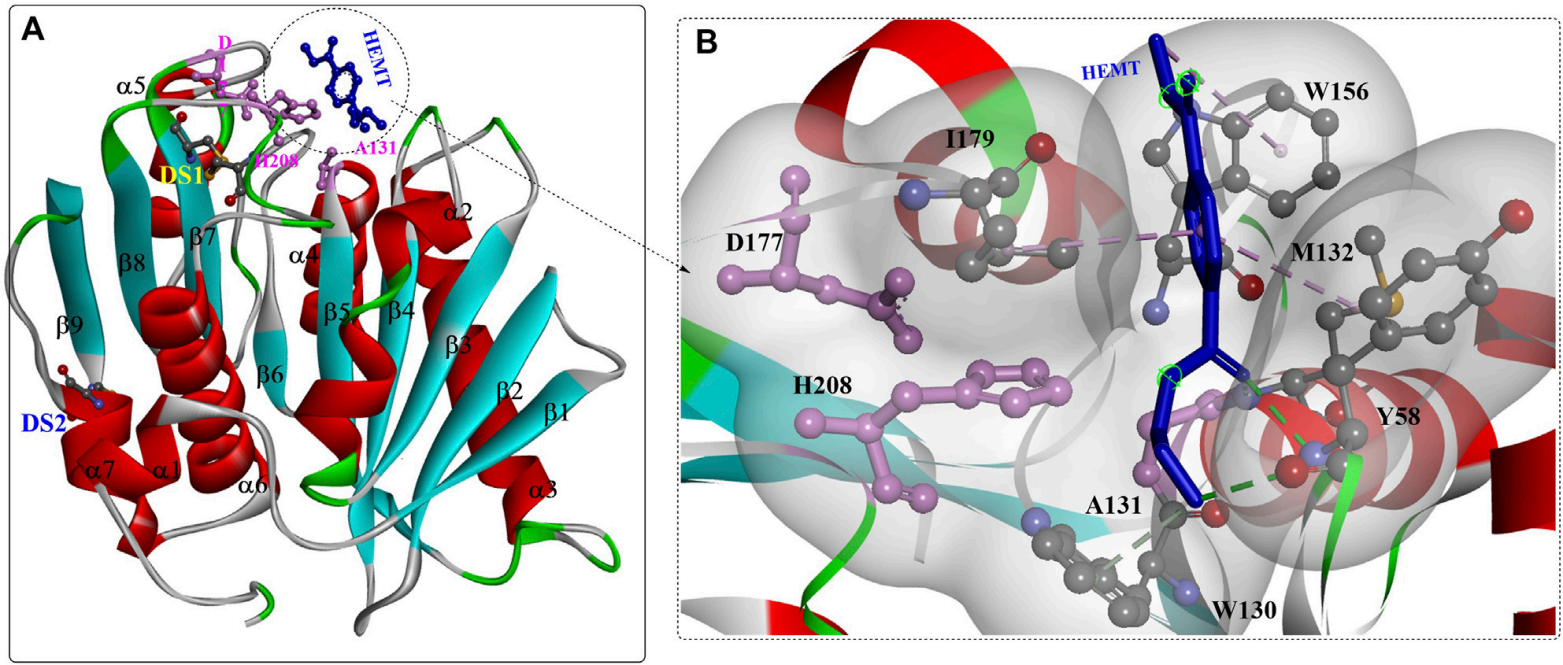
The overall structure of IsPETase (**(A)**, PDB ID: 5XH3) and the interaction modes **(B)**. (The figures in this manuscript were generated using the program Discovery Studio 3.0; the special structural elements are shown in ball-and-stick model; the dotted green (or light green) lines indicate the formed H-bonds; and the pink dotes line mean the hydrophobic interactions. The solid ribbon indicates the protein structure, which are coloured by red [the alpha helix), blue (the β-sheet) and white (the β-turn)].

Based on the structures (Figure 1A) (Han et al., 2017; Joo et al., 2018), *Is*PETase belongs to the α/β hydrolase superfamily with highly conserved structures, including the central twisted β-sheet (α3) formed by 9 mixed β-strands and surrounded by 7 α-helices and the Gly-X-Ser-X-Gly motif. Ser160, Asp206 and His237 form the catalytic triad, and two disulfide bonds (C203-C239 and C237-C289) were also identified. Site-directed mutation analysis showed that DS1, W156, S185 and Y58 might play critical roles in PET degradation (Han et al., 2017). Moreover, the residues W156 adjacent to the active site displayed variable conformations, including the “A”, “B” and “C” conformers, which are important for substrate binding and product release. Interestingly, the enzyme-substrate interactions of *Is*PETase are highly identical to those of ICCG, and the most notable variation is that the aromatic moiety of two bound ligands deviated by ∼30°(Zeng et al., 2022).

The substrate binding site (HEMT) is located in a shallow cleft surrounded by the hydrophobic residues A131, H208, W156, I179, W130, Y58, and M132 on the protein surface (Figure 1B). It was also revealed that *Is*PETase exhibits a more open active-site cleft than homologous cutinases (Austin et al., 2018). In addition, the flexible β1-β2 connecting loop far from the active site was suggested to be responsible for the increased stability and activity of IsPETase, and the β7-α5 connecting loop might be associated with substrate binding (da Costa et al., 2021).

Based on special features and phylogenic analysis, *Is*PETase-like (PL) enzymes and canonical cutinases are divided into type I enzymes, type IIa enzymes, and type IIb enzymes (Joo et al., 2018). Type I enzymes lack the extensive β8-α6 loop and have an extra disulfide bond that is not conserved in the other homologous enzymes. Type IIa and IIb enzymes are characterized by different putative secondary substrate-binding subsites: Ser/Thr is conserved in type IIb enzymes and Phe/Tyr is conserved in IIa enzymes.

**FIGURE 2:**
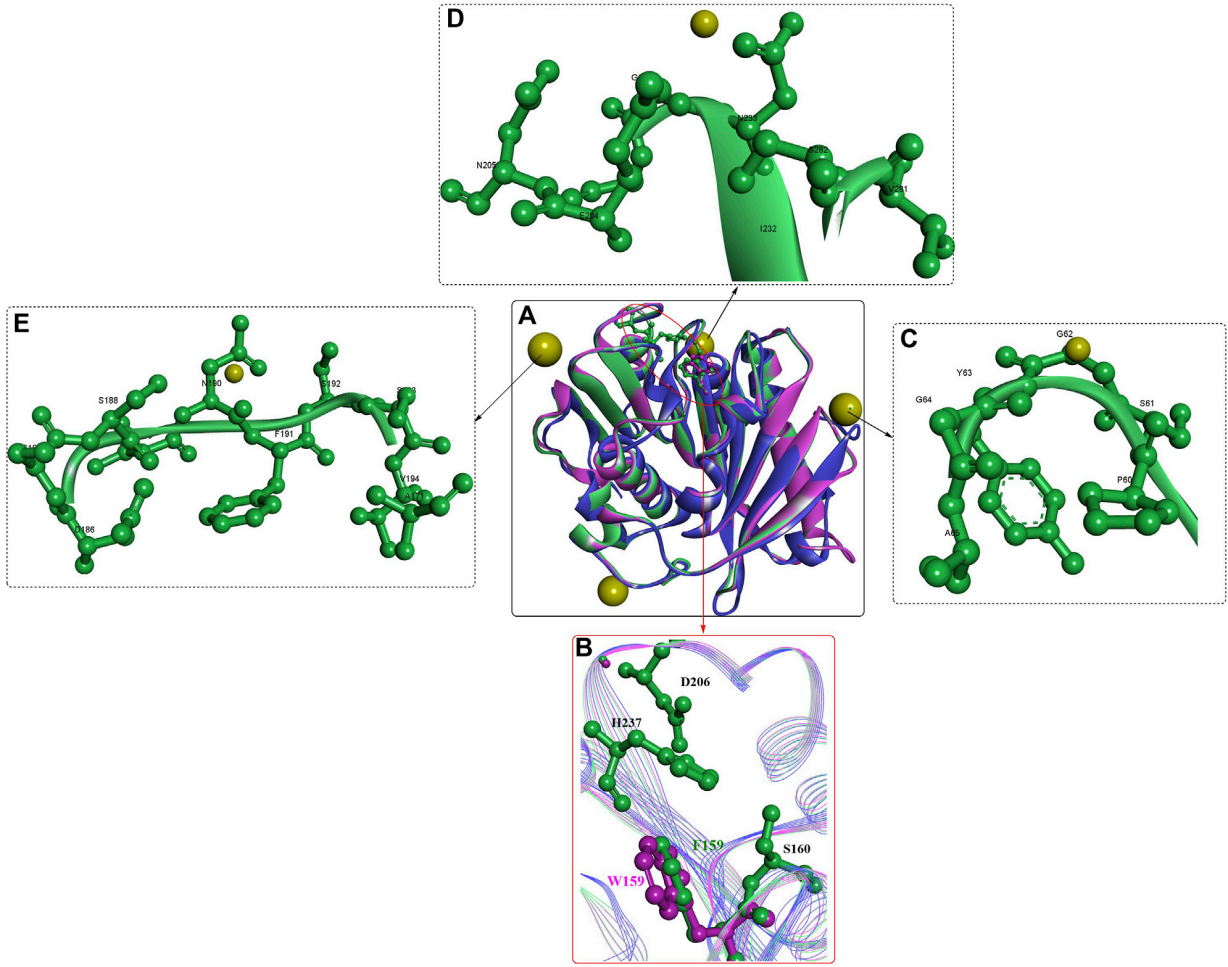
The structure alignment of IsPETase [**(A)**, PDB ID: 6ILX in green, and 6ILW in brown] with cut190 (PDB ID: 5NZO in pink), the active site **(B)** and the Ca^2+^ binding sites **(C–E)**. (The yellow resides mean the bound Ca^2+^).

Further study showed that *Is*PETase was also capable of efficiently degrading p-hexanoate (*p*-NH) esters (k_cat_ = 1,345.46 s^−1^, K_m_ = 0.0528 mM, and k_cat_/K_m_ = 25,487). Structure analysis showed that the crystal structure of *Is*PETase is quite similar to that of cut190, particularly in the similar fold of the Ca^2+^-binding sites (Figures 2A, C–E). Although the promotion effects of Ca^2+^ and ^+^ were revealed in this study, to date, no crystal structure has displayed the binding of Ca^2+^ to *Is*PETase. *Is*PETase displayed the highest enzymatic activity at 35°C; however, at 55°C, it showed almost no enzymatic activity (C. Liu et al., 2019). To improve the thermostability of *Is*PETase, immobilization by ammonium sulfate precipitation and glutaraldehyde cross-linking was performed: consequently, the optimal reaction temperature was increased from 35°C to 45°C, and the enzyme retained ∼60% of the maximum activity at 65°C. Given that hydrophobic and steric effects might be associated with the different substrate specificities, critical residues around the catalytic centre were engineered for altered substrate specificity. The results showed that the mutations S93M, W159F, and IN241F could all change the substrate specificity of wild *Is*PETase with improved enzymatic activities for 1-naphthyl acetate. In particular, the W159F mutant displayed the highest catalytic activity for 1-naphthyl acetate, which might be caused by the mutated Phe contributing to the entrance of the bulky substrate naphthyl to the catalytic cleft, alleviating the steric effect (Figure 2B).

#### 2.1.2 Promising mutants of *Is*PETase

##### 2.1.2.1 *Is*PETase^S121D/D186H^

To date, great efforts have been made to improve the thermostability and PET-degrading activity of *Is*PETase. Stefan Brott et al. applied random mutagenesis with error-prone PCR for the screening of potential mutants with enhanced performance (Brott et al., 2022). The obtained mutant *Is*PETaseTM^K95N/F201I^ showed a 5.0°C higher T_m_ value than the wild type but also had slightly lower activity on PET. Recently, a web-based mutation design tool (Premuse, http://www.elabcaas.cn/pird/premuse.html) was developed to predict preferred mutations (Meng et al., 2021). As a semirational design strategy based on natural sequence evolution, the main feature of Premuse was the pairwise alignment process and the calculated position-specific amino acid probabilities (PSAPs). The former was used to process alignment, avoiding the calculations necessary for multiple sequence alignment, and the latter was used to quantitatively select the preferred mutations. The results showed that the best mutant, W159H/F229Y, was identified from only ten variants, and the Tm, kcat/km and half-life were increased by 10.4°C, 2-fold and 3-fold compared with those of wild *Is*PETase. The mean decomposition rate reached 23.4 mg PET/h/mg enzyme for the PET bottle after 3 days. This study is important for the rational design of promising PET-degrading catalysts; however, no crystal structures have been published highlighting the detailed contributions caused by site-directed mutations.

**FIGURE 3:**
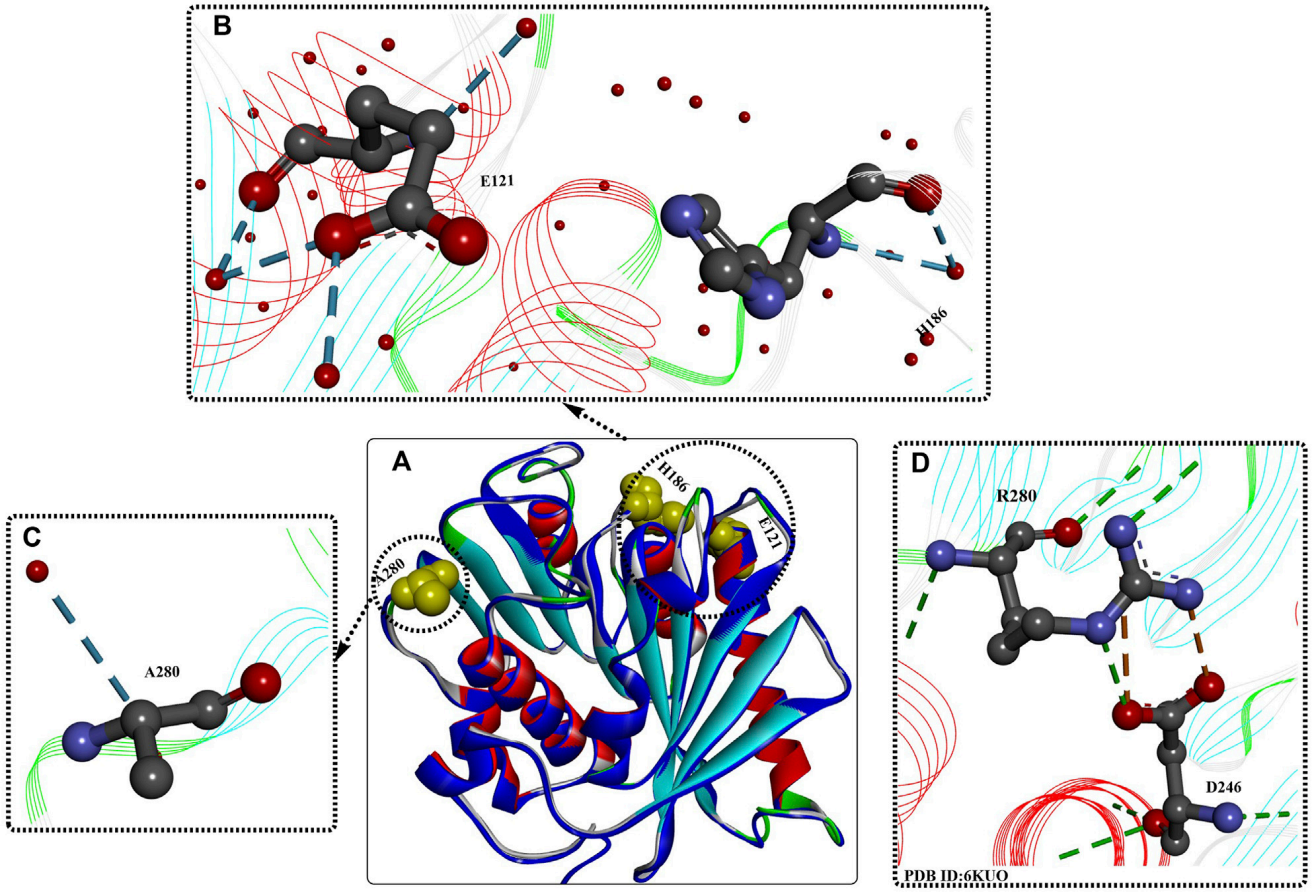
Structure analysis of IsPETase [**(A)**, PDB ID: 8GU5 in blue] and IsPETase^S121E/D186H/R280A^. [**(A)**, PDB ID: 6IJ6 in multiple colours] and the interactions between the mutations **(B–D)**. (The dotted green (or light green, and blue) lines indicate the formed H-bonds; the brown dotes line mean the formed salt bridging interactions; the free red residues mean the water molecules; and the line ribbons indicate the protein model).

As shown by the crystal structures of *Is*PETase (Son et al., 2019), the central β sheet is interrupted by the Pro181 residue located in the abnormal conformation of the β6 strand (Figure 3A), which is believed to play an important role in the thermal stability. Therefore, the P181A mutant was constructed to recover the continuity of the central β sheet. In addition, the B factors show that the β6-β7 connecting loop (Asp186-Phe191) located at the protein surface is one of the most flexible regions in *Is*PETase without any interactions with neighbouring regions. This was considered to be another important factor influencing its thermostability. The mutant S121D/D186H was generated to increase the thermostability of the β6-β7 connecting loop by the formation of an additional H-bond. The results showed that the T_m_ values of *Is*PETase^P181A^ and *Is*PETase^S121D/D186H^ were 49.25°C and 54.85°C, respectively, reflecting increases of approximately 0.5°C and 6°C. The decreased catalytic activity of the P181A, P181G, and P181S mutations indicates that P181 might play an essential role in thermostability and PET degradation ability. This hypothesis must be further tested. However, the *Is*PETase^S121D/D186H^ mutant displayed increased thermostability and PET-degrading activity simultaneously, and at 40°C, the PET-degrading activity of *Is*PETase^S121D/D186H^ was increased by 6.0-fold after 72 h of incubation.

##### 2.1.2.2 ThermoPETase

Based on previous findings (Joo et al., 2018), the synergistic effect of the R280A mutation was integrated into *Is*PETase^S121D/D186H^ and *Is*PETase^S121E/D186H^, further improving the Tm values by ∼7.6°C and 8.81°C, respectively, compared with that of *Is*PETaseWT. Moreover, the PET-degrading activity of *Is*PETase^S121E/D186H/R280A^ was further increased by 13.9-fold compared to that of wild-type *Is*PETase after 72 h of incubation at 40°C. Structural analysis of *Is*PETase^S121E/D186H/R280A^ (ThermoPETase, Figure 3) showed that the R280A and D186H mutations contribute to the formation of 1 and 2 additional H-bonds with the free water molecule, respectively, and the S121E mutation helps form 2 additional intramolecular H-bonds and 2 extramolecular H-bonds with free water molecules. Although the catalytic performance of *Is*PETase^S121E/D186H/R280A^ was significantly improved, including the thermostability and PET-degrading activity, the detailed mechanisms were not clearly elucidated. In this study, the improvements were mainly caused by the increased thermostability and durability.

##### 2.1.2.3 *Is*PETase ^S121E/D186H/N246D/S242T^

In a subsequent study, a structural bioinformatics-based strategy was used for the rational design of *Is*PETase (Son et al., 2020). The study mainly focused on the engineering of the substrate binding site, and based on multiple structure alignment, 27 residues constituting the substrate binding site of *Is*PETase were identified. In addition, 9 residues were further selected for site-directed mutagenesis, and the resulting mutants *Is*PETase^S242T^ and *Is*PETase^N246D^ displayed significantly increased activities. After incubation at 37°C for 72 h, the catalytic activities of *Is*PETase^S242T^ and *Is*PETase^N246D^ were increased by 1.5- and 2.4-fold, respectively, compared with those of wild-type *Is*PETase. However, when the N246D mutation was added to *Is*PETase^S121E/D186H/R280A^, the results showed that the obtained *Is*PETase^S121E/D186H/N246D/R280A^ displayed significantly decreased activity and thermostability. Additionally, from the crystal structures (Figure 3D), salt bridges between D246 and Arg280 and the additional formed H-bonds were identified, which might be factors in the increased catalytic performance. This indicates that the N246D mutation might be more important for enzyme activity. Based on these findings, the S242T mutation was integrated into *Is*PETase ^S121E/D186H/N246D^; the results showed that the Tm value was increased by 1°C, and the PET-degrading activity was increased by 58- and 1.5-fold compared with that of wild-type *Is*PETase and the *Is*PETase^S121E/D186H/R280A^ mutant at 37°C.

##### 2.1.2.4 DuraPETase

In a recent study, a computational strategy, greedy accumulated strategy for protein engineering (GRAPE), was developed to improve the robustness of *Is*PETase (Y. Cui, Chen, et al., 2021b). The GRAPE strategy included three main steps: 1) The generation of stabilizing mutations with multiple algorithms (e.g., ABACUS, FoldX, Consensus analysis) led to the identification of 253 unique predicted mutations. 2) The characterization of beneficial mutations based on biophysical pitfalls led to a sublibrary of 85 candidates. Among them, 21-well-expressed mutants were determined to show increased stability. Based on the *K*-means algorithm, these 21 identified hits were further clustered into three groups for the subsequent accumulation process. 3) Accumulation of the mutations in each cluster according to the greedy algorithm based on the stability-mediated physical features led to a total of 65 combined variants, among which *Is*PETase-M10 (named DuraPETase, S214H-I168R-W159H-S188Q-R280A-A180I-G165A-Q119Y-L117F-T140D) from the third cluster was identified as the most thermostable variant, displaying dramatically increased thermostability. The T_m_ value of DuraPETase was increased to 77°C, which was enhanced by ∼ 31°C compared with wild *Is*PETase.

**FIGURE 4:**
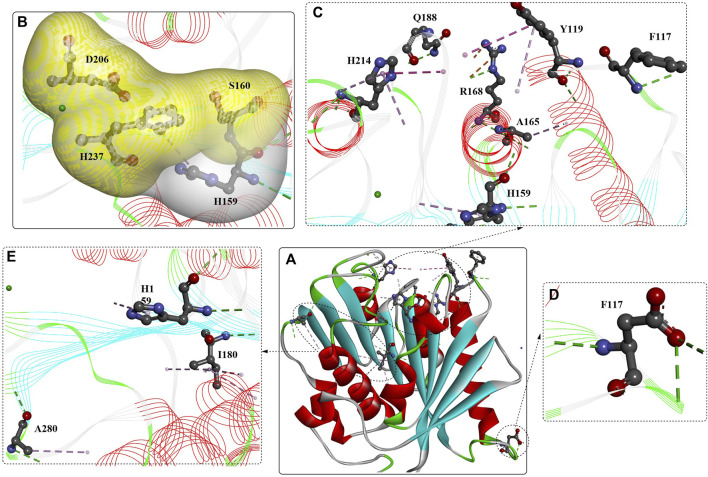
Structure analysis of Dura-PETase [**(A)**, PDB ID: 6KY5] and the interactions between the mutations **(B–E)**. (Meanings of different dotted lines are the same as mentioned above; the free green residues indicate the Ca^2+^; the yellow and white surface indicate the formed hydrophobic regions).

When applied for the digestion of semicrystalline PET film (30%) after 10 days at 37°C, the released soluble products reached up to 3.1 mM, and the conversion was determined to be ∼15 ± 1%, which was ∼300-fold higher than that released by *Is*PETase. Compared with LCC, TfH, Tfcut2, Cut190, and Cut190^Q138A/D250C-E296C/Q123H/N202H^, the catalytic performance of DuraPETase against the same PET film was increased by 1.7-, 13-, 55-, 10-, and 8-fold after 3 days of incubation at 37°C. Importantly, at 37°C, complete degradation of nanoplastics (⌀ = 50–100 nm) was catalysed by *Is*PETase and DuraPETase within 1 h. Structure analysis and molecular simulation showed that the mutations introduced new electrostatic interactions (I168R, shown as orange lines Figure 4C), improved hydrophobic packing [L117F, Q119Y, A180I, S214H, and R280A, shown as pink lines (Figures 4C, E), reduced the conformational entropy (G165A, Figure 15D), and led to the formation of additional H-bonds (shown as green lines).

In particular, the W159H mutation located near the catalytic triad probably reconstructed the binding site of DuraPETase (shown in grey surface, Figure 4B), which is also true for the functions of the L117F and Q119Y mutations via π-π interactions with Tyr87. S214H was concluded to be capable of preventing the wobbling of Trp185 by an offset parallel π-π stacking interaction. Mutations S214H, Trp185, Tyr87, L117F, and Q119Y finely regulate the active-site cleft suitable for PET binding, which was named the “aromatic tunnel” flanker. Tyr87, Q119Y, and Trp185 could enhance PET binding through π-π interactions with the aromatic motif. The incorporation of these mutations might contribute to the increased catalytic performance, which further illustrates the improved ability to lower the likelihood of combining mutations with adverse effects and a lack of target protein structures in enzyme engineering.

##### 2.1.2.5 DuraPETase^N233C/S282C/H214S/S245R^

Based on DuraPETase, another mutant named DuraPETase-4 M (DuraPETase^N233C/S282C/H214S/S245R^) was designed to increase the overall rigidity (N233C/S282C), regulate the flexibility of the W185 region (H214S), and optimize the protein surface electrostatic charge (S245R) (Y. Liu, Liu, et al., 2022a). It should be noted that the H214S substitution led to decreased stability at 60°C, and the degradation performance against the amorphous PET powder was significantly improved. This also reflects the activity-stability trade-offs. When maintained at 60°C for 96 h, DuraPETase-4 M efficiently hydrolysed the PET film (crystallinity 10.2%) with a degradation percentage of 69.8%, which is a 5.4-fold improvement over DuraPETase.

Similarly, an electrostatic interaction-based method was developed for the rational design of *Is*PETase and investigating the roles of electrostatic interactions in thermostability (Yin et al., 2022). Residues S92 (on the α3 helix), I139 (on the α4 helix) and D157 (on the loop connecting β6 and β7) on the protein surface were rationally selected and mutated to the positively charged residues. The I139R mutation led to the highest increase in ΔT_m_ of 8.71°C (T_m_ = 56.37°C), the R251A mutation caused a Tm value increase of 4.68°C (T_m_ = 52.34°C), and the S92R/D157E mutation resulted in an increase of 2.18°C (T_m_ = 51.48°C). Introduction of the R251A mutation into the above obtained mutants further improved their T_m_ values, which were 53.08°C (ΔT_m_ = 5.42°C) and 54.80°C (ΔT_m_ = 7.14°C) for the *Is*PETase^S92K/R251A^ variant and *Is*PETase^S92R/D157E/R251A,^ respectively. With improved thermostability, *Is*PETase^I139R^ also displayed a 3.6-fold improvement in degradation activity. Interestingly, using the MHET substrate at 30°C, the *k*_cat_ of the *Is*PETase^S92K/D157E/R251A^ variant was shown to be increased by 1.74-fold. In particular, the *Is*PETase^S92K/D157E/R251A^ variant could fully degrade PET into TPA alone without MHET, which might be caused by a narrower substrate binding cleft. Thus, only the smaller, EG moiety was capable of entering the active site for further hydrolysis. This is an unusual and important finding, especially for promoting the PET circular economy in the future.

##### 2.1.2.6 FAST-PETase (PETase^S121E/D186H/R224Q/N233K/R280A^)

**FIGURE 5:**
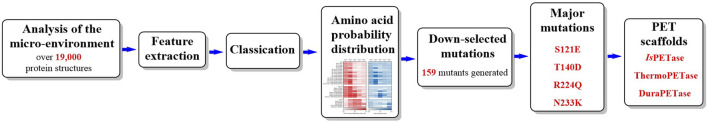
The detailed workflow of MutCompute.

In a similar study, rational protein engineering based on a structure-based machine learning algorithm was applied for the design of robust and efficient PET hydrolase (Lu et al., 2022). MutCompute (https://mutcompute.com), a web-based tool based on deep learning-guided predictions for protein mutagenesis, was used for the discovery of thermostable mutants (Figure 5). From all 29 possible combinations with the four obtained mutations (S121E, T140D, R224Q and N233K) across the three promising PETase scaffolds (*Is*PETase, ThermoPETase and DuraPETase), 23 mutants displayed increased Tm compared with their respective scaffolds. In particular, FAST-PETase (PETase^S121E/D186H/R224Q/N233K/R280A^) showed a 38-fold increase in PET hydrolytic activity at 50°C compared with that of ThermoPETase, with 33.8 mM PET monomers released after 96 h. Even compared with LCC and LCC^ICCM^, FAST-PETase also demonstrated better performance at mild temperatures and moderate pH conditions. In particular, at 50°C, FAST-PETase displayed the highest overall depolymerization rate of the highly crystalline substrates compared with the thermophilic enzymes (LCC and ICCM), *Is*PETase and ThermoPETase and DuraPETase. Moreover, FAST-PETase is capable of depolymerizing the pretreated films (∼2% crystallinity) by quick temperature quenching, releasing 32.8 mM PET monomers at 50°C after 24 h. It could even degrade highly crystalline (23.6%) films by releasing 23.8 mM PET monomers.

**FIGURE 6:**
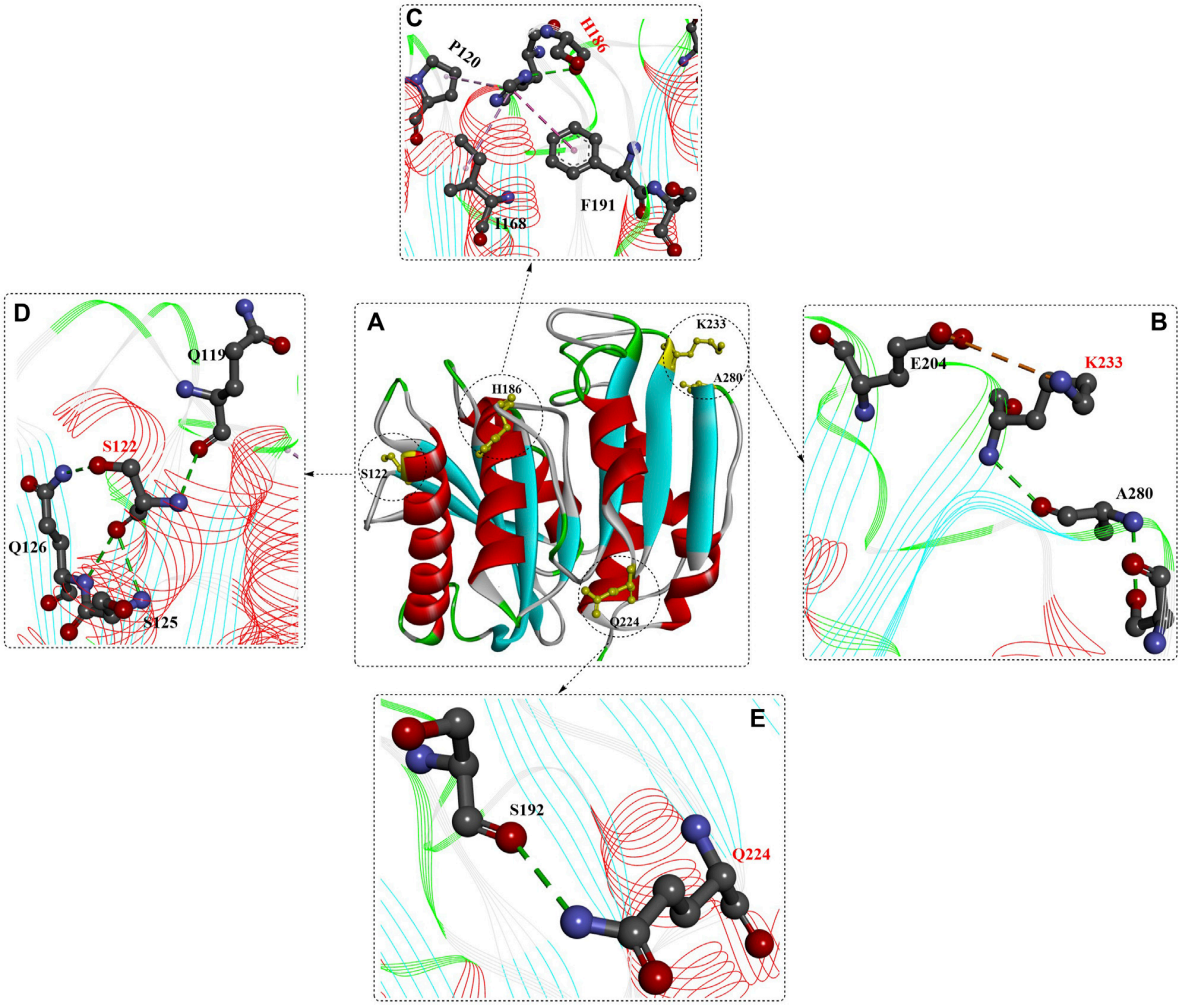
Structure analysis of FAST-PETase (PDB ID: 7SH6) and the interactions of different mutants with favourable residues [(**B–E**), the red residues mean the mutant residues]. (Meanings of different dotted lines are the same as mentioned above).

Structure analysis (Figure 6) revealed several additional intramolecular salt bridges (shown in the orange line formed between the K233 mutation and E204), Figure 6B), hydrogen bonds (Figure 6B–D, shown in green lines) and hydrophobic interactions (shown in pink lines formed between the H186 mutation, P120 and F191, Figure 6C). Importantly, the portability and effectiveness of these machine learning-based predictions were further validated by the introduction of the corresponding N233K mutation to LCC, ICCM and Cut190. The results showed that the ΔT_m_ values of LCC^D238K^ and Cut190^D250K^ were increased by 7°C, although the ΔT_m_ of ICCM^C238K/C283S^ was decreased by 3.7°C on amorphous gf-PET.

Raghav Shroff et al. developed a structure-based deep learning model, a 3D convolutional neural network (CNN), which could be trained to deduce amino acid-structure relationships by connecting amino acids with the local chemical microenvironments (Shroff et al., 2020). This would be helpful for the discovery of novel gain-of-function mutations that might be missed by energetics-based strategies. It empirically identifies native residues discordant with their structural and chemical expectations or the local environment as ideal targets for rational protein engineering. The results indicate that this strategy performs better in combination with the widely used protein design tools based on energetics simulations and generates potential gain-of-function mutants with high-frequency and state-of-the-art accuracy. Ultimately, the obtained mutants with improved function *in vivo* were determined to be increased by approximately 6- and 30-fold across three diverse proteins.

##### 2.1.2.7 HotPETase

**FIGURE 7:**
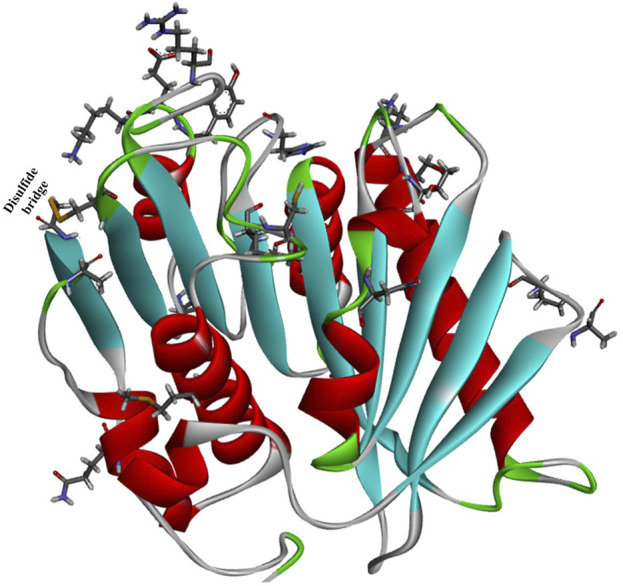
The overall structure of HotPETase (PDB ID: 7QVH) and the mutant amino acids (shown in ball and stick representation).

Robust PETases satisfying the demands of industrial processes are key to realizing industrial plastic biodegradation. To further improve the thermostability of *Is*PETase, an automated, high-throughput directed evolution protocol was reported for the screening and identification of promising PET-degrading biocatalysts (Bell, Smithson, and Kilbride, 2022). The reaction temperature and the extended reaction time were used for selection pressure. After six rounds of evolution, the most thermostable *Is*PETase variant, HotPETase (*Is*PETase^S121E/D186H/R280A/P181V/S207R/S214Y/Q119K/S213E/N233C/S282C/R90T/Q182M/N212K/R224L/S58A/S61V/K95N/M154G/N241C/K252M/T270Q^, Figure 7, PDB ID: 7QVH), was identified. It contains 21 amino acid mutations compared to the wild *Is*PETase, and the *T*_m_ (82.5°C) was increased by 25.7°C compared with the starting *Is*PETase^TS^. At 65°C, after 1 h, each mole of HotPETase releases more monomers than LCC^ICCG^ does, revealing its superior catalytic performance. In particular, under the optimal conditions, 0.5 μM HotPETase could degrade cryPET (0.4% crystallinity, 4 g/L), releasing 6.07 mM soluble monomer within 5 h at 60°C with a degree of depolymerization of 31%. This is the most promising *Is*PETase mutant for PET biodegradation.

Crystal structure analysis showed that the P181V mutation created an additional hydrogen bond, altered surface charge distributions, especially in the substrate binding cleft, and substantially decreased flexibility around the regions formed by the residues from Ala183 to Asn190 and Cys203 to Leu216. The mentioned mutations would further improve the packing of the central β-sheet region; moreover, the well-defined conformation of Trp185 was shown to be unnecessary for efficient PET deconstruction. This finding illustrates the obscure functions of P181 (Son et al., 2019).

##### 2.1.2.8 GrAnc8

In a recent study (Joho et al., 2023), ancestral protein reconstruction (ASR) was used to understand the evolutionary history of *Is*PETase from ancient serine hydrolases. First, the phylogenetic trees and ancestral sequences were reconstructed with the ASR1 dataset (82 sequences, constructed by GRASP) and ASR2 data (147 sequences, constructed by CodeML). Then, the Rosetta-based algorithm protein repair one-stop shop (PROSS) was used for the identification of evolutionarily conserved mutations responsible for stability and catalytic activity. This resulted in a library of 15 PETase mutants containing 9 to >50 mutations, among which 5 mutants (GrAnc9, GrAnc8, CoAnc3, PROSS4, PROSS5) exhibited significantly increased thermostability with T_m_ values approximately 20°C higher than that of the wild *Is*PETaes.

In particular, the mutant GrAnc8 was determined to be the best performing ancestor in terms of stability and activity. Through structure analysis, significant variation in surface-exposed residues was identified, especially those lining the substrate binding site. It was believed that the more cationic trend might cause the improved stability; however, this (the altered surface charge) was determined to be unrelated to any functional adaptation of *Is*PETase. In addition, the deletion of a surface-exposed loop (ADLK) between S278 and T279 was expected to result in a clear increase in thermostability. Moreover, it was shown that a segment around the active site (R171A, N186D, F218I, S214H, D186S) played critical roles in regulating the degradation activity and thermostability by accommodating the conformations of W185. The structural basis for the evolution of the improved activity revealed that it is mainly caused by remote mutations through the increased level of sampling of catalytically productive conformations of the existing functional residues. In this study, the activity-stability trade-offs were also confirmed, as in the case of S214H, D186S and D186N, which increased stability but decreased activity.

#### 2.1.3 MHETase

Attention has been attracted again by the MHETase for PET degradation, accompanied by the rapid development of *Is*PETase for the efficient conversion of mono-(2-hydroxyethyl) terephthalate (MHET) to terephthalic acid (TPA) and ethylene glycol (EG) with k_cat_ = 31 ± 0.8 s^−1^ and K_m_ = 7.3 ± 0.6 mM. Compared with *Is*PETase, there are far fewer reported studies on the structures, functions and engineering of the MHETase enzyme.

In the host *I. sakaiensis* 201-F6, the MHETase gene (ISF6_0224) is located adjacent to the TPA-degrading gene cluster (Yoshida et al., 2016), and the protein sequence shows sequence similarity to those of the tannase family with highly conserved catalytic triad residues. However, MHETase is determined to be nonhomologous to the 6 determined MHET-hydrolytic enzymes (TfH, FsC, Thc_Cut1, Thc_Cut2, Thf42_Cut1, *Bs*EstB), which was also confirmed by the fact that MHETase was unable to degrade PET, BHET or pNP aliphatic esters catalysed by the tannase.

**FIGURE 8:**
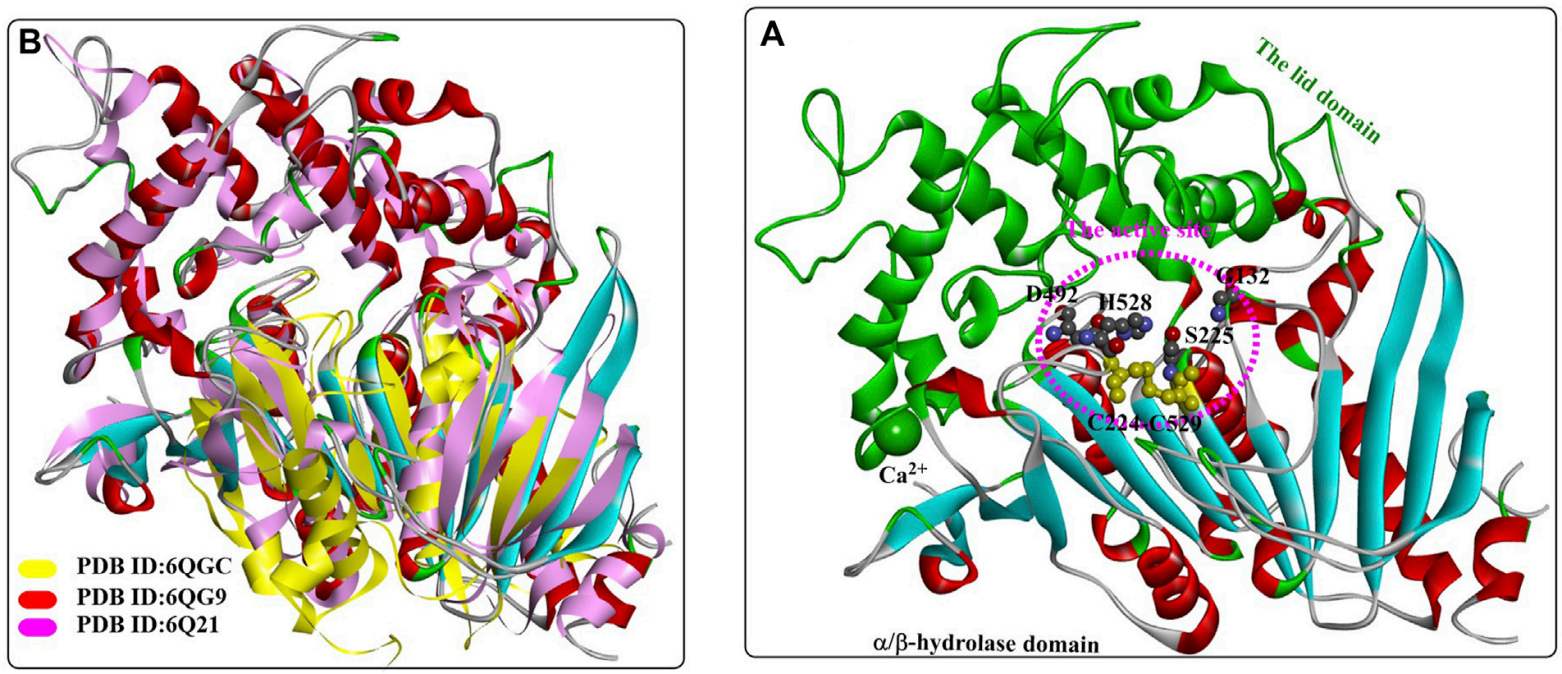
The overall structure of MHETase **(A)**, (PDB ID: 6QG9) and the structure alignment **(B)** of MHETase, an esterase from Aspergillus oryzae (PDB ID: 6Q21) and the IsPETase from *Ideonella sakaiensis* (PDB ID: 6QGC). (The active site of MHETase is highlighted with pink dotted lines, and the lid domain are coloured by green and the α/β-hydrolase domain is coloured by red (the alpha helix), blue (the β-sheet) and white (the β-turn).

Weber et al. reported the crystal structure of the active ligand-free MHETase from *I. sakaiensis* bound to a nonhydrolyzable MHET analogue (Palm et al., 2019). The overall architecture of MHETase is more similar to that of feruloyl esterases (Figure 8B), and it possesses the classic α/β-hydrolase domain, the highly conserved catalytic triad S225-H528-D492, one highly conserved disulfide bond (C224-C529) among five internal disulfide bonds (C51-C92, C224-C529, C303-C320, C340-C348, C577-C599), and the presence of a Ca^2+^-binding site with the critical residue G132 in the oxyanion hole and the lid domain. The disulfide bond can stabilize the catalytic residues Ser and His, and the lid domain associated with substrate specificity might be further maintained by the bound Ca^2+^ (Suzuki et al. 2014).

**FIGURE 9:**
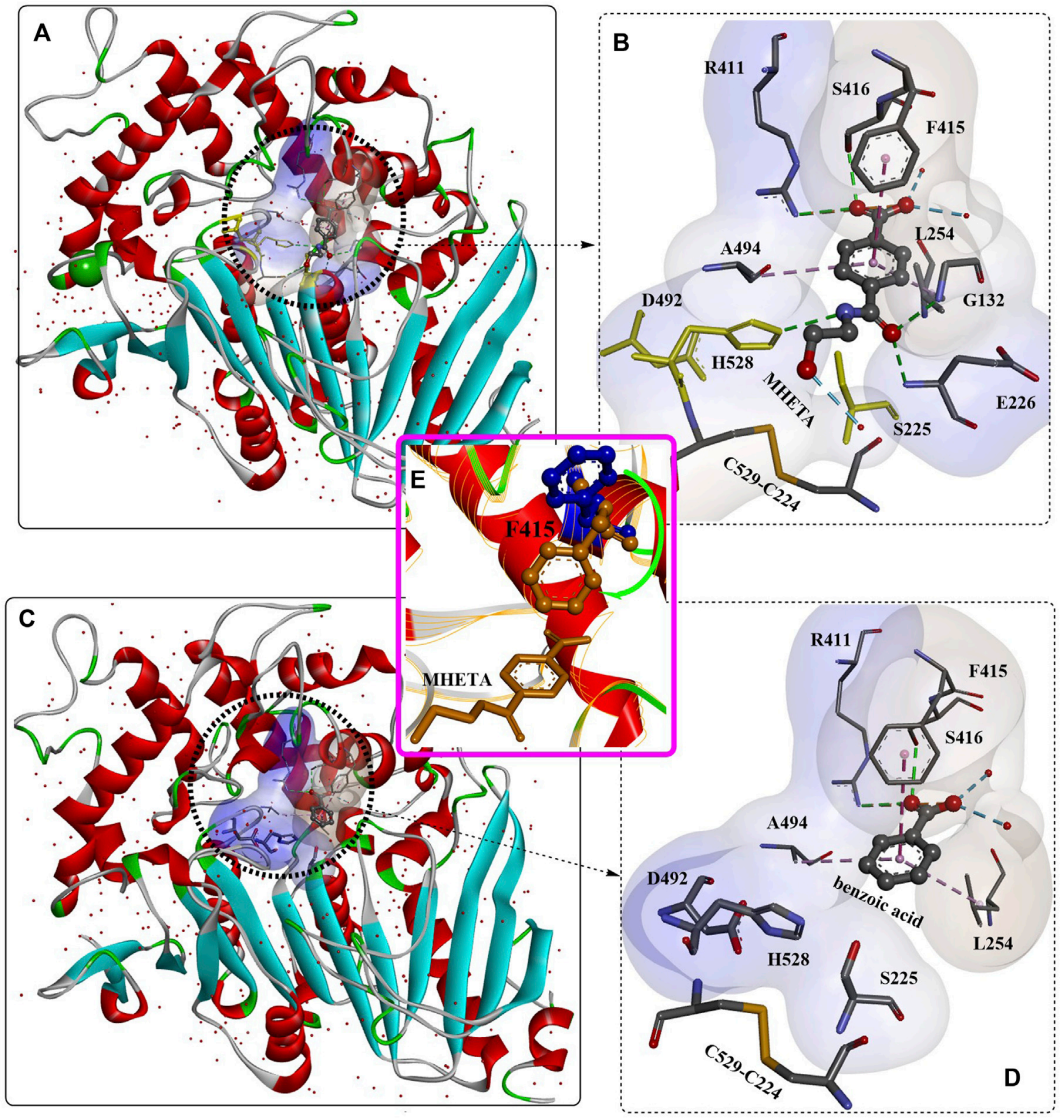
The interaction modes of MHETase with a nonhydrolyzable ligand MHETA **(A, B, E)**; PDB ID: 6QGA and with benzoic acid **(C, D)**; PDB ID: 6QGB. (The light blue surface indicates the substrate-binding site).

The binding mode of the MHETase-MHETA complex (Figures 9A, C) revealed that the substrate specificity is almost entirely conferred by the lid domain. In the substrate binding site, the substrate MHETA is stabilized by the hydrophobic interaction between the phenyl ring of MHETA and the residues F495, G132 and A494 (Figure 9B). Furthermore, the free carboxylate is tightly bound by the lid domain residues R411 and S416 and two water molecules through H-bond interactions. In addition, the peptide bond maintains a hydrogen bond network with H528, G132 and E226. In particular, F415 functions as a “gatekeeper” that is positioned away from the active site when the enzyme is ready for substrate binding; once the substrate is bound, the side chain of F415 is triggered to rotate by nearly 180°, closing the gate. This probably leads to much tighter interactions between MHETase and the substrate with a K_m_ of 7.3 μM. The functions of R411 were further investigated by site-directed mutagenesis, and the results showed that R411A and R411Q almost completely lost their MHET hydrolysing activity. This is also true of S416 or S419 for regulating substrate binding. Moreover, R411A, R411Q and F424N, with altered positive charges, displayed significantly higher hydrolysing activity against BHET, indicating altered substrate specificity.

**FIGURE 10:**
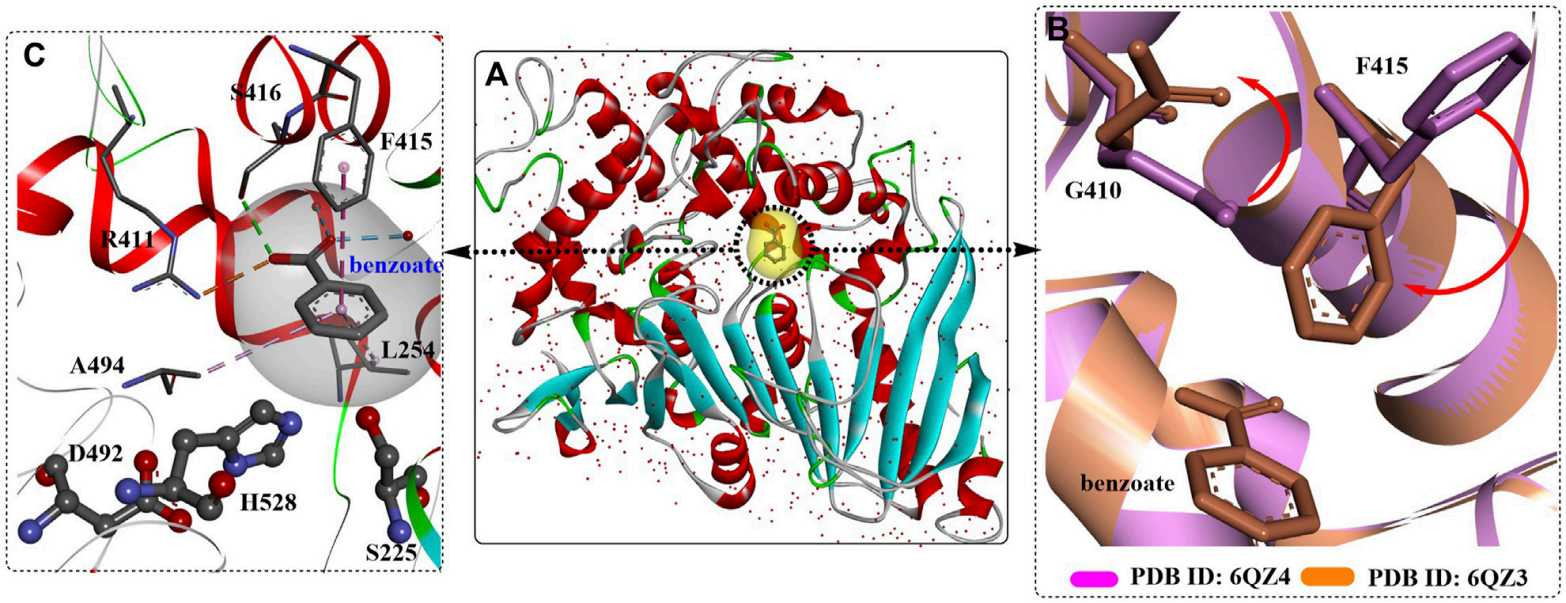
The crystal structure of MHETase with benzoic acid **(A)**, PDB ID: 6QZ3), the detailed binding modes of benzoic acid **(C)** and the special conformation caused by substrate binding **(B)**. (The substrate-binding site is coloured by yellow; and the red arrows mean the orientation of the conformational changes).

Recently, a 1.6 Å resolution MHETase structure was reported (Figure 16) (Knott et al., 2020), which also highlights the MHETase core domain, the lid domain and a Ca^2+^ binding site, similar to the overall structure in Figure 10A. As discussed above, distinct structural conservation is also revealed between the MHET-hydrolysing domain of MHETase and *Is*PETase. Except for the observed F415 adopting a “closed” orientation on substrate binding, the side chain of G410 would also trigger the active site with ligand binding (Figure 10B).

In addition, molecular simulation indicated that the lid could be further stabilized upon Ca^2+^ binding, and the two-step reaction (acylation and deacylation) mechanism was also investigated. First, catalytic S225 is activated and deprotonated by H528, making it ready for nucleophilic attack upon the carbonyl C of MHET, which will lead to the formation of released, EG and the acyl-enzyme intermediate. It is worth noting that the deacylation reaction proceeds without, EG in the active site, and the nucleophilic attack on the AEI by a water molecule liberates TPA. This process was also assisted by H528, which could deprotonate the catalytic water and transfer the proton to the catalytic S225 for another catalytic cycle. Biochemical studies showed that MHETase also displayed concentration-dependent substrate inhibition with a *K*_m_ value of 23.17 ± 1.65 μM and a *K*_k_ value of 307.30 ± 20.65 μM. Although MHETase^S131G^ showed little concentration-dependent substrate inhibition, it displayed an 8-fold higher *K*_m_ value and an ∼3% reduction in catalytic activity, indicating a poor affinity for MHET.

The catalytic performance of the lidless MHETase was also explored, which led to a significantly decreased catalytic rate (∼1,000-fold lower, *k*_cat_ = 0.05 ± 0.03 s^−1^). This demonstrates the important role of the lid domain in MHET-hydrolysing activity. The inability of MHETase to act on MHE-isophthalate (MHEI) and MHE-furanoate (MHEF) was also detected. Considering the synergistic effect on PET depolymerization, chimeric enzymes were constructed with flexible glycine-serine linkers (8–20 glycine and serine residues) connecting the C-terminus of MHETase to the N-terminus of PETase. The results showed that the chimeric proteins all exhibit markedly improved PET and MHET turnover compared with the single free enzyme or the mixed two free enzymes. In my opinion, this finding is quite important for the design of promising and effective multienzyme systems for the efficient degradation of PET waste.

**FIGURE 11:**
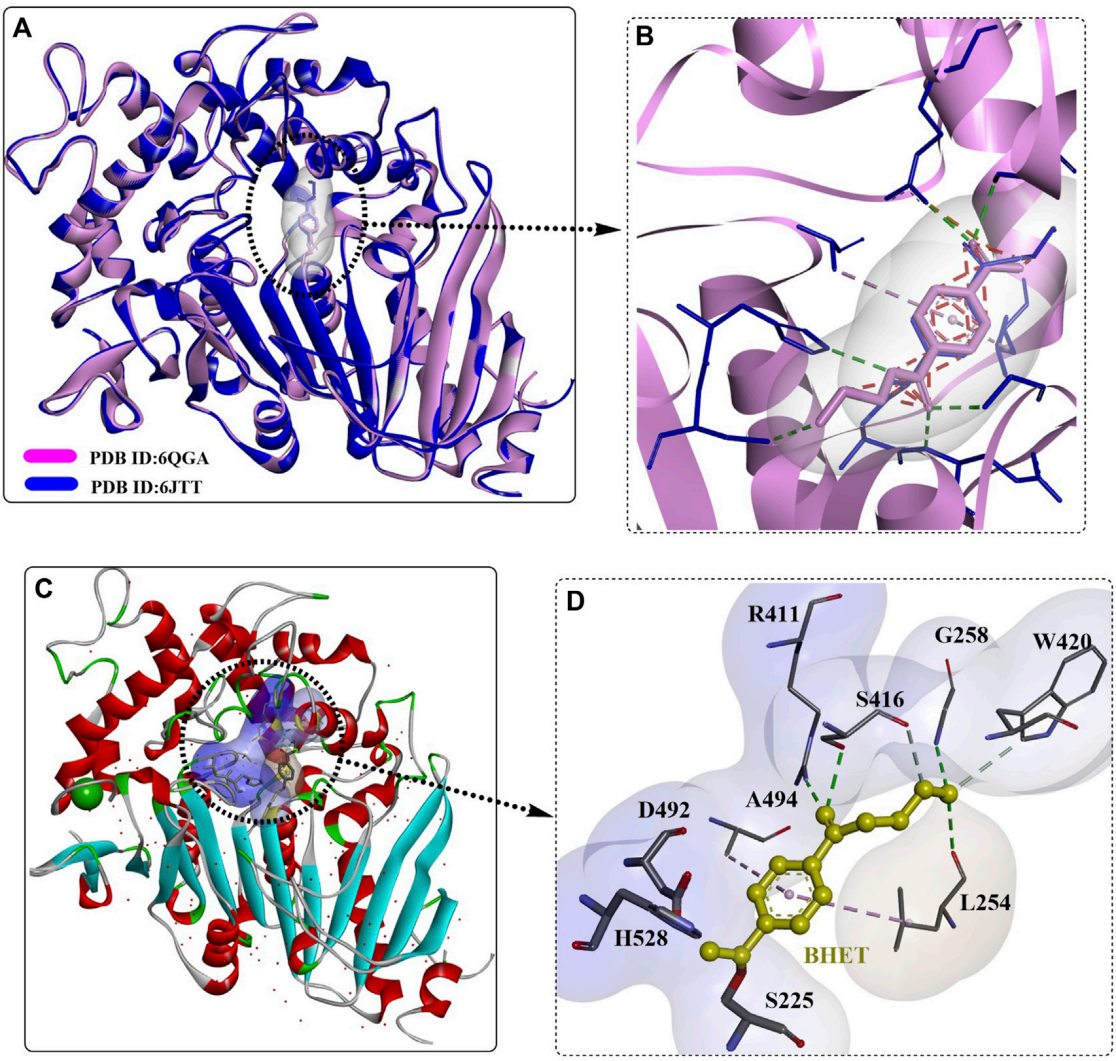
The structure alignment of MHETase with different ligands **(A, B)**, the detailed structure of MHETase with BHET **(C)**, PDB ID: 6JTT) and the interaction modes **(D)**. (The light blue and white surface indicates the substrate-binding site; the free red residues indicate the water molecules; and the green residues mean Ca^2+^).

The secretory expression of MHETase in an active form with proper folding was achieved by Kim et al. in *Escherichia coli* with the SP_LamB_ system (Hye Young Sagong et al., 2020). It was found that MHETase might exist as a monomer, in contrast to the structural homology of the dimer feruloyl esterase from *Aspergillus oryzae*. This was believed to be caused by the different conformation of its lid domain, which could inhibit subsequent dimerization. The overall structure of the secreted MHETase is almost identical to the released structure and functions in an enzyme concentration-dependent manner. Crystal structure analysis showed that the binding modes of MHET and BHET in the active site of MHETase are quite similar to each other (Figures 11A, B); in addition, the TPA moiety was found to form an ester bond with the catalytic S225 residue. This indicates that BHET could also be hydrolysed by MHETase, which was further confirmed to function in a concentration-dependent manner although with a significantly lower hydrolysing activity. The BHET was bound to the active site lined by R411, S416, G258, W420, A494, L254, D492, H528, and S225. The, EG moiety was further stabilized by R411, S416, G258 and L254 through H-bonds, and the TPA moiety was also regulated by hydrophobic interactions with A294 and L254. The special conformation of the, EG moiety indicates that MHETase might also display *exo*-PETase activity capable of hydrolysing the terminus of PET, which was confirmed by the successful hydrolysis of PET_5_ to TPA.

Given these, rational engineering of the MHETase for improved BHET-hydrolysing activity was achieved. First, MHETase^R411K^ was designed to add a salt bridge with the carboxyl group of MHET to stabilize the ester bond. The protein was obtained and determined to show a 1.7-fold increase in activity compared with that of wild-type MHETase. Second, to remove the steric hindrance effect caused by F424 located in the inner substrate-binding site and to provide effective space and potential hydrogen bonding partners for BHET binding, MHETase^F424N^, MHETase^F424V^, and MHETase^F424I^ were constructed, and the BHET-hydrolysing activities were increased by ∼3.9-, 3.0-, and 3.4-fold, respectively. Combined with the R411K mutation, the obtained double mutants (MHETase^R411K/F424N^, MHETase^R411K/F424V^, and MHETase^R411K/F424I^) all displayed improved catalytic activity, especially the MHETase^R411K/S416A/F424I^ variant, which exhibited a 15.3-fold increase in BHET hydrolysis activity. At the same time, MHETase^R411K/F424V^, MHETase^R411K/F424I^, and MHETase^R411K/S416A/F424I^ exhibited significantly enhanced degradation activity against the PET5 oligomer compared with wild-type MHETase. Although the obtained mutants displayed no hydrolysing activity against the amorphous PET film without a terminus, wild-type MHETase showed degradation activity against the PETase^S121A/D186H/R280A^-treated PET film, and MHETase^R411K/S416A/F424I^ displayed even higher hydrolysing activity. The BHETase activity and the *exo*-PETase activity of MHETase were further confirmed by these results.

### 2.2 Other novel PET hydrolases

#### 2.2.1 PHL7

**FIGURE 12:**
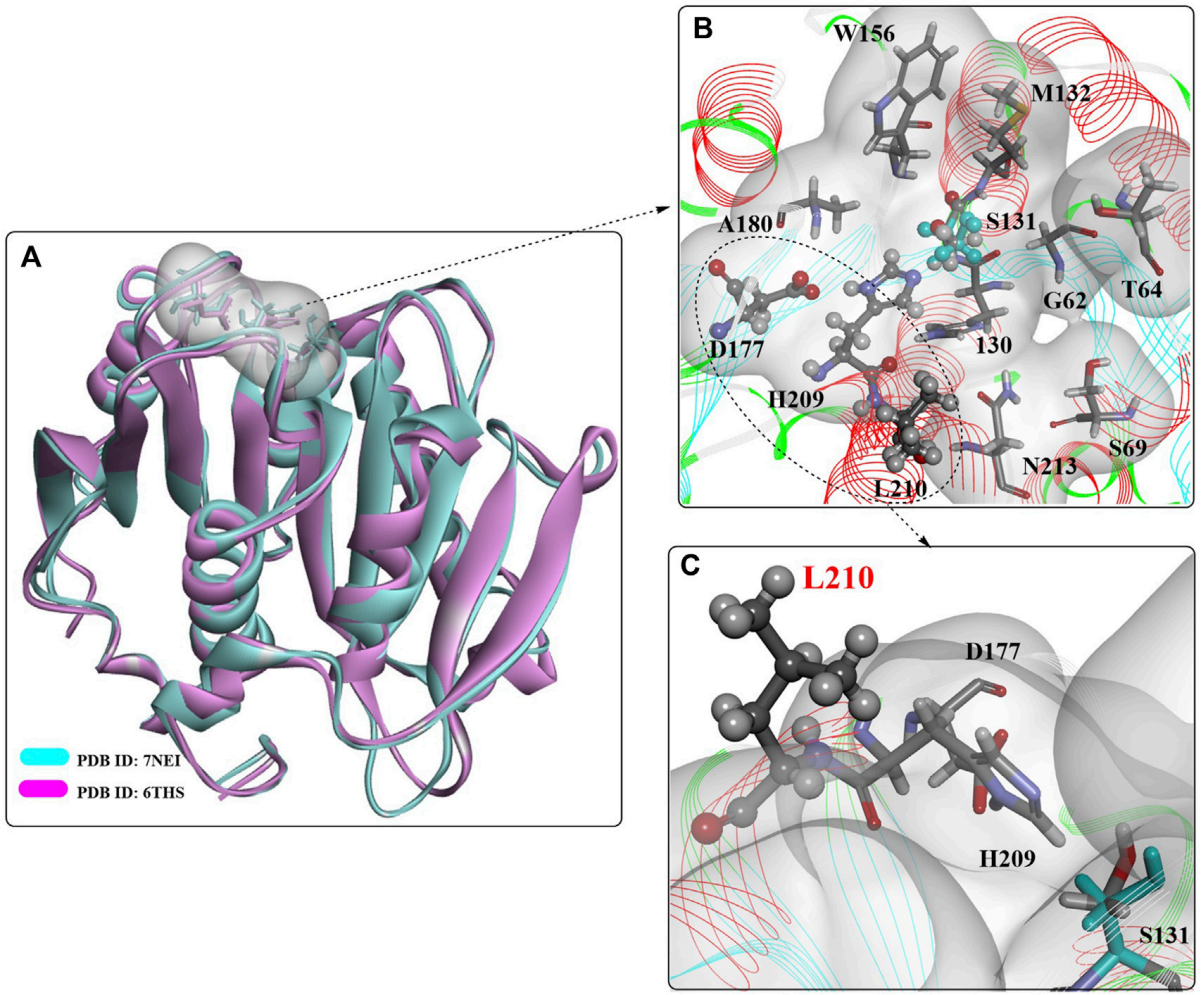
The overall structure of PHL7 **(A)** and the interaction modes **(B,C)**. (The white surface indicates the substrate-binding site).

A novel PET hydrolase (PHL7, ENA accession number: LT571446, PDB ID: 7NEI) was isolated from plant compost and is capable of degrading amorphous PET films and postconsumer PET with high efficiency (Sonnendecker et al., 2022). The overall structure of PHL7 is almost the same as that of LCC^S165A^ (PDB ID: 6THS, Figure 12), displaying the typical features of the α/β-hydrolase fold superfamily with a highly conserved Ser-Asp-His catalytic triad. It is believed that the similar overall crystal structure and substrate-binding cavity would lead to similar catalytic mechanisms. Importantly, the active site pocket of PHL7 is more open than that of LCC, mainly because of the different corresponding residues F63, L93, Q95 and I179. This difference might be helpful for the binding of larger PET substrates and could be a key to more tolerant recognition and effective processing of PET at elevated temperatures concomitant with increased polymer chain mobility.

#### 2.2.2 BgP

Dobson et al. screened 51 marine bacterial isolates for the discovery of potential ester/polyester hydrolytic catalysts, in which *Brachybacterium ginsengisoli* B129SM11 was identified from the deep-sea sponge *Pheronema sp*. as a potential PET-degrading polyesterase producer (Carr et al., 2022). Sequence analysis combined with a genome “mining” strategy (Almeida et al., 2019) revealed a putative PET hydrolase (named BgP) that shares high sequence similarity with TfCut2 (62%). Bioinformatics analysis showed that 54% of the residues in BgP are identical to Cut190; moreover, 83% of the amino acid residues displayed similar biochemical properties to those of Cut190, such as highly conserved features (e.g., α/β fold). A single disulfide bond (Cys287 and Cys302) was found near its terminal end by structural analysis, similar to type I PETase. In addition, Tyr62, a hydrophobic residue, plays a critical role in the stabilization of the intermediate, and two Ca^2+^ binding sites were identified at positions 31 and 37 (site 1 in Cut190) and positions 158 and 160 (site 3 in Cut190).

#### 2.2.3 *Rg*PETase

**FIGURE 13:**
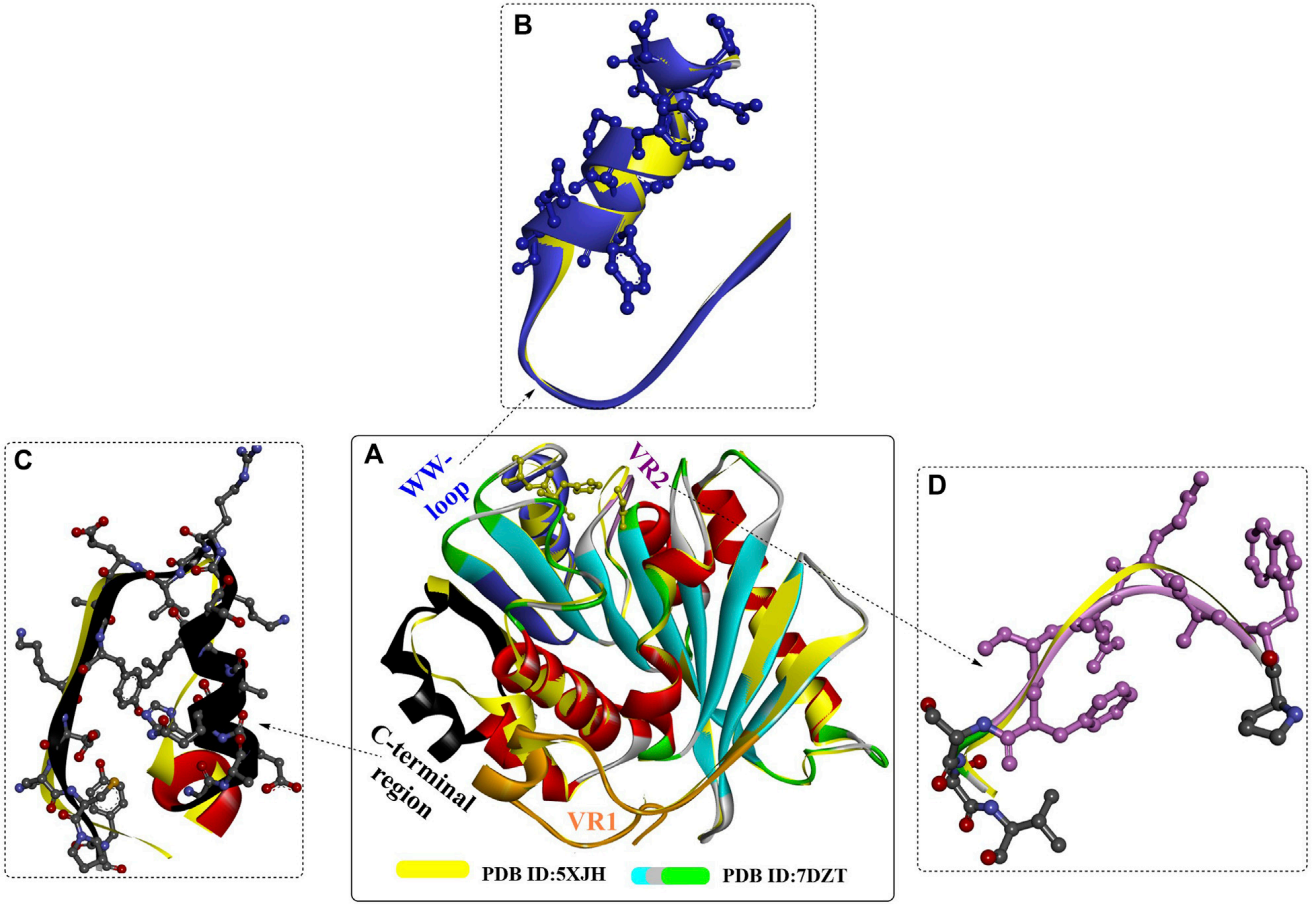
The structure alignment of RgPETase with IsPETase **(A)** and their structural differences **(B–D)**.

A novel PET hydrolase from *Rhizobacter gummiphilus* (*Rg*PETase) was identified (T_m_, 48.5°C), which displays almost the same degrading activity against microcrystalline PET but a significantly lower activity compared to that of *Is*PETase at 30°C (H. Y. Sagong et al., 2021). Crystal structure analysis indicated that *Rg*PETase shares the key structural features of *Is*PETase, such as the catalytic triad, substrate binding cleft, extended β8-α6 connecting loop, and two disulfide bonds (Figure 13A), which might lead to high PET hydrolysis activity. However, *Rg*PETase also displays substantial structural differences (Figures 13B–D), including variable region 1 (VR1, Q26-S51), the wobbling tryptophan-containing loop (WW-loop, W183-S187), variable region 2 (VR2, P207-V228) and the C-terminal region (G271-Y292). These special structures might contribute to the different behaviours of *Rg*PETase toward low-crystalline PET. In particular, the increased activity of *Rg*PETase^K169A^ and the decreased activity of *Rg*PETase^E186A^ and *Rg*PETase^K169A/E186A^ showed that the electrostatic charge of the WW-loop surface or the whole protein surface of *Rg*PETase might greatly affect its PET-hydrolysing activity.

#### 2.2.4 SM14est

Recently, an *in silico*-based screening (sequence similarity search) approach using BLASTP was reported for the screening of PETase-like enzymes from the genus *Streptomyces* (Almeida et al., 2019). From a total of 52 genomes analysed, 3 potential PETase-like enzymes from marine sponge-associated *Streptomyces* isolates were successfully identified, including SM14est from *Streptomyces* sp. SM14. Sequence analysis revealed that the serine hydrolase motif (G-X1-S-X2-G) and the catalytic triad (Ser-Asp-His) are also conserved in SM14est. Heterologous expression of SM14est was achieved in *E. coli* with the native *Streptomyces* signal peptide, which was also confirmed to show polyesterase activity by the polycaprolactone (PCL) plate clearing assay. With a similar method, a cascade of BLAST searches was applied to identify potential PETases in the Joint Genome Institute (JGI) terrestrial metagenome datasets (7,375 samples) (Karunatillaka, Jaroszewski, and Godzik, 2022).

#### 2.2.5 *Bb*PETase

In recent studies, a novel PET hydrolase from *Burkholderiales bacterium* RIFCSPLOWO2_02_FULL_57_36 (*Bb*PETase) was identified and characterized with an additional N-terminal domain (*Bb*PETase^AND^) (H. Y. Sagong et al., 2022; C.C. Chen Xsy et al., 2021). The T_m_ values of *Bb*PETase^Full^ (the full-length form) and *Bb*PETase^CD^ (the N-terminal truncated form) were 54°C and 51°C, respectively. These changes might be responsible the improved PET hydrolysis activity of *Bb*PETase^Full^, and after 3 days, this mutant still displayed 3.5-fold (1.9-fold for *Bb*PETase^CD^) higher activity than that of wild-type *Is*PETase at 40°C. However, it should be noted that although *Bb*PETase^AND^ might play a role in the improved thermostability of *Bb*PETase, this domain also showed a detrimental effect on PET hydrolysis. Especially for the low-crystallinity PET film, *Bb*PETase^Full^ and *Bb*PETase^CD^ both exhibited lower activities than *Is*PETase at 30°C. This might be caused by *Bb*PETase^AND^ inhibiting the adhesion of PETase binding to the PET surface. Based on these findings, the increased thermostability might be caused by the additional N-terminal domain, which led to the improved catalytic activities.

**FIGURE 14:**
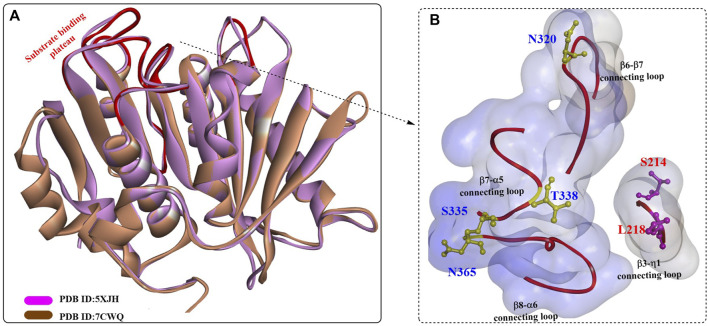
The structure alignment of BbPETaseCD with IsPETase **(A)** and the substrate binding cleft **(B)**. (The blue surface indicates the substrate-binding site, and the red line highlight the residues associated with formation of the substrate binding plateau).

To date, the crystal structure of *Bb*PETase^Full^ has not been released, but the detailed structure of *Bb*PETase^CD^ was obtained recently (PDB ID: 7CWQ, Figure 14) (C.C. Chen et al., 2021). It is almost identical to that of *Is*PETase and shares most of the key structural features of *Is*PETase, such as the canonical α/β-hydrolase fold and the substrate-binding site. Although the substrate-binding cleft was similar, the different residues on the surface led to slightly different conformations and surface charge distributions. The L218, S220, and T338 residues caused *Bb*PETase^CD^ to form a flatter and more hydrophobic site, and the S335, M363, and N365 residues resulted in a deeper and narrower substrate-binding cleft. The single mutants S335N, T338I, M363I, and N365G and the multipoint mutant *Bb*PETase^S335N/T338I/M363I/N365G^ (*Bb*PETase^NIIG^) were constructed, and the results showed that the Tm of *Bb*PETase^NIIG^ was increased by 9°C and the PET-degrading activity was increased by 2.8-fold at 40°C.

However, the crystal structures of *Bb*PETase^AND^ or *Bb*PETase^Full^ have not been obtained; therefore, the detailed interactions between *Bb*PETase^AND^ and *Bb*PETase^CD^ might be inaccessible. However, it is certain that the two domains could directly interact with each other. Moreover, it was shown that S214 and I218 in *Is*PETase exert a major influence on its higher PET-degrading activity at ambient temperature and substrate specificity, which are associated with W185 wobbling and β6-β7 loop flexibility (C.C. Chen et al., 2021). Sequence analysis revealed that S214 and I218 of *Is*PETase are consistently replaced by His and Phe, respectively, in the other homologues. This special role was further confirmed by the improved PET-degrading activities of several *Is*PETase-like enzymes with site-directed mutations of His/Phe residues to Ser/Ile.

Although *Bb*PETase shows similar PET-degrading activity against microcrystalline PET, it displays higher thermostability than wild *Is*PETase. Based on detailed structural comparisons between *Bb*PETase and *Is*PETase, we generated the BbPETase^S335N/T338I/M363I/N365G^ variant with enhanced PET-degrading activity and thermal stability. We further revealed that *Bb*PETase^AND^ contributes to the thermal stability of the enzyme through close contact with the core domain, but the domain might hinder the adhesion of the enzyme to the PET substrate.

#### 2.2.6 PE-H

**FIGURE 15:**
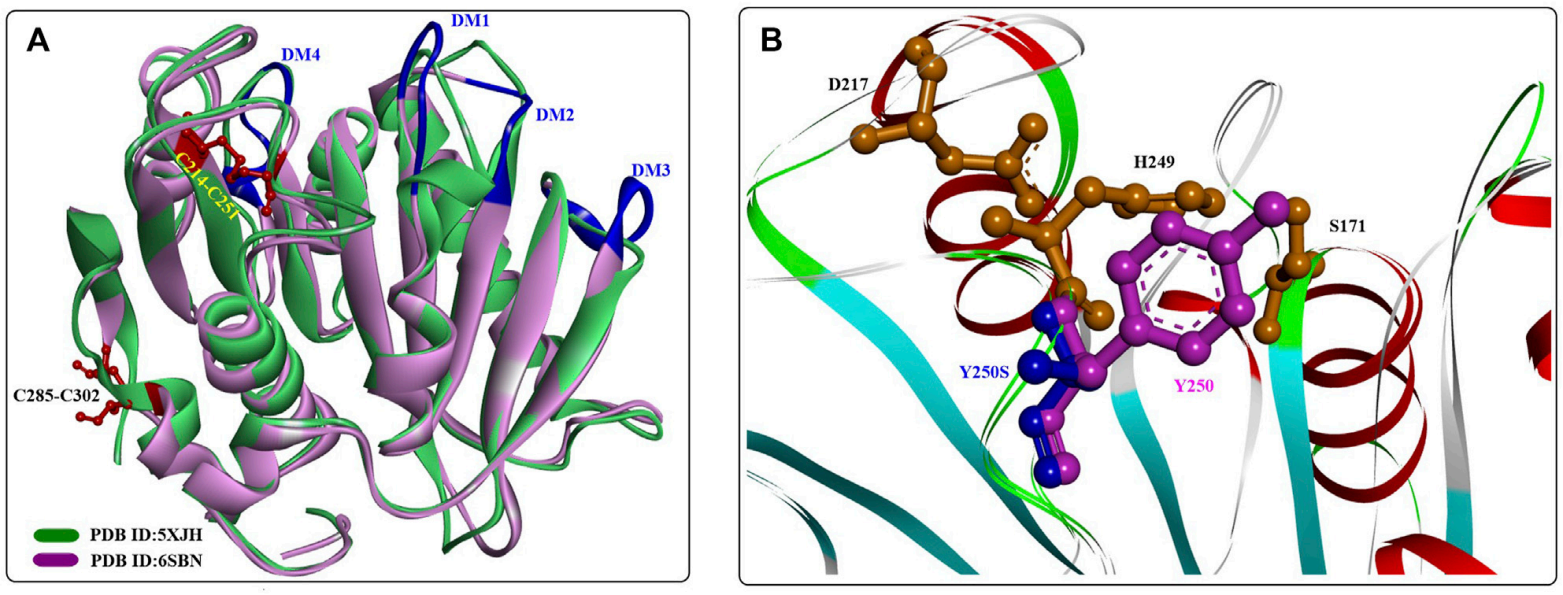
The structural alignment of PE-H with IsPETase **(A)** and the main structural characteristics **(B)**. (The dark blue lines indicate the special loop, and the formed disulfide bonds are coloured by red in **(A)**].

A novel polyester hydrolase (PE-H) from the marine bacterium *Pseudomonas aestusnigri* was identified as belonging to the type IIa family of PET hydrolytic enzymes determined by sequence alignment (Bollinger et al., 2020). Similar to the overall structures of wild *Is*PETase, PE-H (Figure 15) is also characterized by a canonical α/β-fold containing a central twisted β-sheet composed of 9 β-strands flanked by 7 α-helices. In addition, 2 disulfide bonds were identified in C214-C251 and C285-C302. The main differences lie in the following four domains (shown in Figure 15, in dark blue): the loop connecting β3-α2 (DM1), the loop connecting β4-α3 (DM2) positioned parallel to it, the loop connecting β1-β2 (DM3), and the loop connecting β6-β7 (DM4). The positioning of DM1 and DM2 away from the active site cleft might create more space for substrate binding.

Considering the effect of Y250 on stabilizing the active site cleft, a Y250S mutant was constructed that has a much deeper substrate binding cleft. The results showed a significant improvement in the PET-degrading activity of PE-HY250S even against the PET bottle, which led to a 45-fold reduction in the amount of MHET released with the PET bottle as the substrate. Although the crystal structures of PE-H and PE-H^Y250S^ (PDB ID:6SCD) are the first reported type IIa PET hydrolytic enzymes, it would be difficult to illustrate the detailed catalytic mechanisms without structures of the ligand‒protein complex.

#### 2.2.7 Mle046

Additionally, in the bacterium *I. sakaiensis*, a novel homologue of the MHETase, Mle046, was recently identified (Meyer-Cifuentes and Öztürk, 2021). Mle046 is also a mesophilic enzyme that is able to rapidly degrade MHET to TPA and, EG at 20°C–40°C. At 30°C, the *K*_m_ and *k*_cat_ values were determined to be 2,638 ± 797 mM [higher than that of *Is*MHETase, (7.3 ± 0.6 mM)] and 80.9 ± 15.8 s^−1^, respectively. This indicates that it has a lower affinity to MHET. In addition to MHET, it could also degrade 4-(4-hydroxybutoxycarbonyl) benzoic acid (Bte), which displayed similar catalytic activity (degradation rate, 0.82 mM s^−1^) to that of MHET (degradation rate, 0.78 mM s^−1^). Moreover, Mle046 showed significant stability over a broad range of temperatures (10°C–60°C) and pH values (6.5–9).

## 3 Strategy for improved PET-biodegradation

### 3.1 Cell surface display for *Is*PETase

In an interesting study, *Is*PETase and the corresponding anchoring hydrophobin (HFBI, GenBank KU173825) were simultaneously displayed on the yeast cell surface for the efficient biodegradation of highly crystallized PET (hcPET) (Z. Chen, Duan, et al. 2022b). The hydrophobicity of the yeast cells could be greatly increased by the displayed HFBI, which would then result in the obvious attachment of the engineered cells with *Is*PETase onto the PET surface (∼2-fold increase in the binding numbers of the engineered cells to PET). The results showed that the catalytic performance of the engineered whole-cell biocatalyst was profoundly improved, and hcPET (crystallinity of 45%) was dramatically increased by ∼328.8-fold compared with wild *Is*PETase at 30°C, with a corresponding conversion level for hcPET of ∼10.9% at 30°C within 10 days.

The special catalytic mechanism makes MHETase much more operational in the biodegradation of PET waste. In a study, engineered *Saccharomyces cerevisiae* expressing the MHETase enzyme was used to develop a whole-cell catalyst for efficient bioconversion of MHET into TPA and, EG (Loll-Krippleber et al., 2022). Efficient MHETase expression with different display partners on the yeast cell surface was achieved, and MHETase-Aga2 and MHETase-Sed1 showed the highest displayed fraction protein and MHETase-Aga2 displayed the protein abundance (4.8 nM for 10^8^ cell/mL suspensions). The MHETase whole-cell biocatalysts showed clearly different catalytic activities, and MHETase-Tip1 displayed the highest reaction rate against MpNPT. MHETase-Tip1 could also efficiently hydrolyse MHET, releasing TPA, and after 24 h, all MHET (400 nM) was converted to TPA. However, no TPA was catalysed by the intracellular MHETase chimaera even after 24 h, which indicates that MHET was hydrolysed extracellularly. However, lower substrate affinity and catalytic efficiency (with a 15.7-fold greater k_m_ and a 0.68-fold lower k_cat_) were also observed and concluded to be caused by ectopic glycosylation, the special environment of the yeast cell surface, or large protein fusion. In contrast, the constructed whole-cell catalysts were confirmed to be active for more than 12 days and stay active up to 45°C.

Recently, a hydrophobic cell surface display (HCSD) system in *E. coli* was developed for the construction of an efficient whole-cell PET-degrading catalyst (Jia et al., 2022). In this method, the FadL protein was used for the display of PETase; moreover, the hydrophobic protein HFBII was displayed on the surface of *Escherichia coli* for increased PET accessibility. The results revealed that the HCSD showed a clear degrading effect on PET, leading to much rougher surfaces with greater hydrophilicity and a water contact angle of 68.4 ± 1°, which was significantly lower than that of untreated PET (106.1 ± 2°). In addition, the HCSD system displayed satisfactory thermostability and processing stability, with 73% activity retained at 40°C for 7 days and 70% activity maintained after 7 cycles. Compared with whole-cell immobilization, the HCSD system exhibits more advantages for efficient PET degradation.

In addition to the widely used model host *E. coli*, *Pichia pastoris* (*P. pastoris*) has long been considered one of the most versatile platforms for the production of various heterologous proteins, including industrial enzymes. Recently, a whole-cell biocatalyst was developed by displaying PETase on the surface of *P. pastoris* to improve the PET-degrading efficiency (Z. Chen, Wang, et al., 2020b). The results showed that compared with free PETase, the obtained whole-cell biocatalyst had enhanced pH and thermostability. Moreover, the turnover rate of the whole-cell biocatalyst against hc-PET was significantly improved by ∼36-fold compared with that of free PETase. In particular, the catalytic performance remained stable after 7 repeated uses, even against commercial hc-PET bottles.

### 3.2 Engineering of *Is*PETase through different secretory expression systems

Given the appearance of abundant N- and O-linked glycosylation sites in *Is*PETase, it is interesting to investigate whether posttranslational glycosylation participates in the catalytic activity and thermostability of *Is*PETase. In one study, it was shown that *Is*PETase could be successfully deglycosylated by endo-β-N-acetylglucosaminidase H (Endo H, EC3.2.1.96), and the thermostability of the recombinant protein (*Is*PETase-*Pp*) could be further elevated. The optimal temperature was increased to 50°C higher than that of wild *Is*PETase; moreover, it retained ∼90% of its initial activity after a 2-h incubation at 50 °C (Deng et al., 2023). The partially deglycosylated *Is*PETase-*Pp* (further deglycosylated by Endo H after Ni-NTA affinity chromatography purification) was determined to be stable at 40°C with no activity lost after heat treatment for 12 h, and it retained more than 40% of its initial activity at 50°C after heat treatment for 6 h. These results demonstrated the higher thermostability of *Is*PETase-Pp compared to the wild-type *Is*PETase expressed by *E. coli* (*Is*PETase-*Ec*). In addition, the catalytic activity of *Is*PETase-*Pp* was further increased by ∼3-fold (from 20.6 U/mg to 62.4 U/mg) after deglycosylation, which was also much higher than that of *Is*PETase-*Ec* (∼1.8 U/mg). Structure analysis showed that N-linked modification sites were mainly detected at N114, N138, N212, N264 and N288 on the loops of the protein surface. After Endo H cleavage, the long polysaccharide chains were removed from *Is*PETase-*Pp,* leaving a GlcNAc residue, which might play critical roles in the increased thermostability.

Although it is widely accepted that the covalent binding of glycans to the protein surface could further improve the thermostability and catalytic stability of the target protein, the mechanism of the enhanced activity of glycosylated and partially deglycosylated *Is*PETase-*Pp* has not been clearly elucidated (Shental-Bechor and Levy, 2008). It should be noted that the substrate-binding sites of PET-degrading enzymes are usually located on the protein surface, and bulky N-linked glycans probably inhibit the interactions between the substrate and the enzymes.

In another study (Kosiorowska et al., 2022), it was shown that *Y. lipolytica* could also be used for the efficient heterologous expression of *Is*PETase, and the engineered host was able to degrade PET film directly with the release of ∼23 mg L^−1^ MHET after 96 h in culture. Moreover, the results showed that 1% olive oil addition could greatly improve the PET hydrolysis efficiency, and the highest concentration of released TPA reached 124 mg L^−1^. It was also found that the addition of 2.5 mM MnSO_4_ alone or 1 mM CuSO_4_ alone could significantly improve the hydrolysis of PET; however, no positive effects on the degradation process were confirmed when the cultures contained 1 mM and 2.5 mM MnSO_4_, 1 mM CuSO_4_, and 1% olive oil. Furthermore, it was highlighted that *Yarrowia lipolytica* was capable of assimilating ethylene glycol and accumulating TPA with PET powder as the substrate.

The secretion of *Is*PETase from *E. coli* has been achieved by using sec-dependent signal peptides (SP), such as the maltose/maltodextrin binding periplasmic protein (SP_MalE_), maltoporin (SP_LamB_) (Seo et al. 2019), and SP_PelB_ (Shi et al. 2021). With SP_LamB_, 6.2 mg of *Is*PETase per 1 L of culture medium was produced, and ∼1.7-fold more secretion of *Is*PETase was achieved by engineered SP_PelB_ via random mutagenesis. In another study, *E. coli* BL21 (DE3) was also selected for increased secretion of *Is*PETase by SP engineering (L. Cui, Qiu, et al., 2021a). Three SP enhancers, B1 (MERACVAV), B2 (MDKACVAV) and B3 (MAERACVAV), were rationally designed and fused to the N-terminus of the widely used SP (PelB, MalE, LamB, and OmpA) to mediate the excretion of *Is*PETase. The results revealed that B1, B2 and B3 could all dramatically promote the secretion of *Is*PETase in *E. coli*, among which SP_B1PelB_ displayed the strongest potential. Compared with that of PelB, the excreted *Is*PETase was increased by 62-fold, and the highest concentration was ∼650 mg/L when induced by IPTG and L-arabinose. The hydrolysis of PET film by crude and purified *Is*PETase was also enhanced after treatment for 42 h, and the released water-soluble products were 58.3 mg/L and 41.5 mg/L, respectively. Moreover, the release of water-soluble products of *Is*PETase catalysis increased by 2.7-fold when the PET film was preincubated with the hydrophobin HFBII. This study provided an easy and convenient method for the biodegradation of PET with the widely used host *E. coli*.

In a similar study, a higher-throughput PETase engineering platform was developed (Zurier and Goddard, 2023), which includes secretory expression and fluorescent product detection. First, YebF-mediated PETase secretion (N-terminal YebF fusion) enables near-parallel expression in microplates, and then, ferric iron oxidation (50 mM FeSO_4_ +500 mM EDTA, pH 8.5) of PET-degrading products results in fluorophores (BHET-OH, MHET-OH, and TPA-OH) that enable rapid activity quantification. Therefore, it could not only eliminate the lysis and purification steps but also permit high-throughput screening of potential PETases. With this method, a library of 54 single point mutations with predicted effects was constructed and screened for positive mutants, which led to a sublibrary of 12 single-point mutants displaying significantly improved PET-degrading activity. It was estimated that the increased activities might be caused by the increased overall stability through the additional intramolecular interactions. Then, the 12 identified positive mutants were combined into 57 double mutants for the screening of higher PETase activity in the next round. This led to the discovery of 9 double mutants showing synergistic effects with significantly higher T_m_ values. Finally, a combinatorial approach was used to optimize PETase activity with the obtained multimutants for 10 days.

The results revealed that SR-PETase (*Is*PETase^A47S/T51A/A74P/V134T/G139N/R280L/N288R^) showed the highest thermostability, released 1.9-fold more degradation products, and had ∼7.4-fold higher activity than wild-type *Is*PETase at 40°C after 10 days. This clearly demonstrates the practicability of applying this high-throughput platform for efficient PETase engineering. In a similar study by K. Liu et al. (2023), a dual fluorescence assay was developed for high-throughput screening for potential PETases. In this method, mutants are first expressed in deep 96-well plates as fusion proteins with a deep red fluorescent protein (mCarmine), which is beneficial to the rapid quantitative assay of enzyme concentrations in a 96-well plate (Plate 1). Second, a specific 96-well plate (Plate 2) was prepared by coating mixed PET fluorogenic probes (FPs) on the bottoms of each well. Finally, transferring an appropriate amount of crude cell lysate from Plate 1 to Plate 2 facilitated the degradation of PET along with rapid cohydrolysis of freed FP to green fluorescein as an indicator of PET hydrolysis, which could then be quantified and determined. With this method, a library of 2,850 mutants with an average of 1-7 amino acid substitutions constructed by error-prone PCR was screened, and the results revealed that through two-round screening, 6 improved variants with 1.3–4.9-fold improved activities were successfully identified. Although slightly complicated, this screening strategy provides another practical alternative for the discovery of potential PETases.

To further improve the soluble expression levels for the efficient production of *Is*PETase, cytoplasmic overexpression in *E. coli* was also investigated (Son et al., 2019). In addition, strategies of coexpression with chaperones GroEL/ES and N-terminal NusA-tag fused expression were also explored in *E. coli* (Aer et al., 2022). The results showed that GroEL/ES could significantly facilitate the overexpression of soluble *Is*PETase^Mut^ at 25°C and 30°C, with the highest improvement of ∼12.5-fold observed at 25°C. This is also true for the superior soluble expression ability of NusA at 25°C, with the highest increase in soluble expression reaching ∼4.6-fold. Although NusA-*Is*PETase^Mut^ and *Is*PETase^Mut^ (GroEL/ES) displayed little improvement in thermostability, NusA-*Is*PETase^Mut^ showed an ∼ 1.5-fold improvement in enzyme activity at pH 9.0. After 2 weeks, a 1.4-fold higher release of soluble TPA, MHET, and BHET (with a much higher amount of MHET) catalysed by NusA-*Is*PETase^Mut^ was obtained in the biodegradation of PET.

As mentioned above, it has been shown that electrostatic interactions play an important role in the protein thermostability of PETase. Recently, an electrostatic interaction-based strategy was developed for the rational design of *Is*PETase with improved catalytic performance, especially thermostability. Based on structural analysis, the domains with high B-factor and RMSF (with higher flexibility) were selected for site-directed mutations, including the positions lined with W156-N161 and E245-R251. The strategy consists of three main steps, including the introduction of a positive charge (S92R and S92K), the substitution of a negative charge (S92R/K + D157E, I139R/K + D157E), and the introduction of the R251A mutation. The results showed that *Is*PETase^I139R^ displayed the highest thermostability with a Tm value of 56.37°C, which was suggested to be caused by reasonable electrostatic interaction with a favourable N-O distance (∼2.7 Å).

When applied for the biodegradation of high-crystallinity PET (hcPET) at 30°C, *Is*PETase^S92R^ and *Is*PETase^S92K/R251A^ displayed better catalytic performance, and the PET-degrading activities were improved by 1.4-fold and 2.5-fold, respectively. The hydrolytic activity of *Is*PETase^S92K/R251A^ was further improved by 3.6-fold at 40°C. In particular, it was found that the only PET hydrolysis product catalysed by *Is*PETase^S92K/D157E/R251A^ was TPA, and after 72 h, the TPA yield was increased by 3.02-fold compared with that of the wild type. Additionally, *Is*PETase^S92K/R251A^ and *Is*PETase^I139R^ displayed distinctly improved thermostability and retained over 60% relative activity for 24 h of inactivation at 40°C. Molecular simulations confirmed that the added electrostatic interaction could efficiently stabilize the target flexible region and lead to global structural stabilization. Especially at higher temperatures, this effect becomes more significant (Schwaminger et al., 2021).

### 3.3 Immobilization of PETase enzymes

Recently, an interesting bienzymatic cascade system (DuP-M@CaP, 1.5 μm) was constructed for the coimmobilization of DuraPETase and MHETase through the SpyTag/SpyCatcher system (K. Chen, Dong, and Sun, 2022c). SpyTag/SpyCatcher (Spy) chemistry shows great potential in multienzymatic cascade reactions with improved catalytic performance, in which SpyTag can spontaneously form stable *iso*-peptide bonds with SpyCatcher. In this study, MHETase-SpyCatcher was first embedded in calcium phosphate nanocrystals (CaP) via biomimetic mineralization, and then DuraPETase-SpyTag was conjugated on the surface. DuP-M@CaP displayed significantly improved thermostability and pH stability; at 50°C, the residual enzyme activity of DuP-M@CaP wase maintained at 87.7% after incubation for 4 h. After 6 days for PET degradation, the degrading efficiencies of DuP-M@CaP were increased by 9.7-fold and 5.2-fold at 40°C and 50°C, respectively, and in a 10-day degradation of PET film, 50.3% weight losses were achieved at 50°C. As expected, the improved stabilities and the specific spatial distribution of the immobilized enzymes contributed to the superior catalytic performance of DuP-M@CaP.

### 3.4 Enhancing PET hydrolytic activity with fused functional domains

The increased accessibility of PET-degrading enzymes towards the highly hydrophobic surface of PET is also important for rapid and efficient PET hydrolysis (C.C. Chen, Dai, et al., 2020a). In addition to modulating protein evolution, the strategy of constructing fusion proteins has also been proven to be effective in enhancing the binding affinity of enzymes and substrates with hydrophobic surface binding modules (D. Ribitsch et al., 2013; D. Ribitsch et al., 2015; Várnai et al., 2014).

#### 3.4.1 Carbohydrate-binding module (CBM)-fused PETase

Carbohydrate-binding modules (CBMs) are small noncatalytic protein domains that usually function as a part of modular enzymes (e.g., carbohydrate-active enzymes, CAZymes) and are capable of enhancing the binding affinity between enzymes and substrates (Armenta et al., 2017; Weber et al., 2019). Consequently, CBMs have been used as efficient affinity tags for protein immobilization owing to their high adsorption capacity for solid substrates (Oliveira et al., 2015; Zhou et al., 2020). Based on the topology of the ligand-binding site, CBMs can be classified into types A, B (*endo*-type), or C (*exo*-type) (Gilbert, Knox, and Boraston, 2013). Type A CBMs display a high binding capacity to crystalline polysaccharides (e.g., cellulose and chitin) through hydrophobic interactions regulated by conserved aromatic triplets and are therefore considered potential candidates for PET-binding peptides (Zhang et al., 2013; Y. Liu, Wang, et al., 2022b).

Joanna et al. reported the identification of a novel PET-binding CBM, and the CBM-PET interactions were explored (Weber et al., 2019). First, a semiquantitative PET surface affinity assay was developed to detect CBMs bound to PET films. Then, eight CBMs from the carbohydrate active enzymes database (http://www.cazy.org/, CAZy database) (Drula et al., 2022) were screened for PET binding, and the results showed that *Ba*CBM2 (GenBank accession numbers: ACQ50287/MK349005) possessed the strongest affinity towards PET. Molecular dynamics (MD) simulations were used investigate the CBM-PET interactions and identified an aromatic triad (Trp9/Trp44/Trp63) on the peptide surface that was also stabilized by π-stacking interactions and hydrogen bonds. This was further verified by tryptophan quenching experiments and alanine point mutations; moreover, the strength of PET binding of CBMs was found to be largely determined by the ratio of hydrophobic to polar contacts at the interface.

Andrew et al. investigated the binding of *Ba*CBM2 for PET in detail and showed that the binding affinity is highly associated with the temperature and crystallinity (Rennison, Westh, and Møller, 2023). With increasing in tested temperatures, the general trend of *K*_d_ increased first and then declined. At ∼40°C, a maximum *K*_d_ of 408 nM for *Ba*CBM2 was detected, showing an obviously lower affinity for this PET substrate. At low temperatures, the increased binding affinity might be caused by a gain in entropy caused by dehydration of the binding surface; nevertheless, at higher temperatures, it might result from the increase in crystalline regions on the PET surface. At 20°C, there is a clear trend of higher affinity at higher crystallinity, and a significant increase in affinity was seen as ∼ 20% bulk crystallinity. This indicated that *Ba*CBM2 might bind preferentially to crystalline areas on the PET surface. MD studies indicated that the binding is mainly driven by the critical residues W9, W44 and W63 through a gain in entropy caused by the dehydration of the binding surface. In my opinion, fusing a polymer-binding module to the PET hydrolase is a promising strategy to streamline the enzymatic hydrolysis of PET, however the binding capacity, affinity, and thermostability should be improved to satisfy the needs of industrial operation.

To improve the PET-degrading capacity of *Is*PETase, different polymer-binding domains (CBM of cellobiohydrolase I from *Trichoderma reesei*, PBM, and HFB4) were selected and fused to the C-terminus of the *Is*PETase^S121E/D186H/R280A^ (*Is*PETase^EHA^) mutant (Dai, Qu, Huang, et al., 2021b). The results demonstrated that CBM fused to *Is*PETase^EHA^ showed the most significant stimulatory effect on the enzymatic hydrolysis of PET with 30.7 μM soluble products (TPA and MHET) released, which was increased by ∼71.5% at 30°C. In contrast, the catalytic activity was found to be strongly inhibited by the fused PBM (poly (3-hydroxybutyrate)–binding domain of PHB depolymerase from *R. pickettii*). However, at 40°C, only *Is*PETase^EHA^_CBM showed improved catalytic efficiency with a total amount of 251.5 μM released products (increased by ∼44.5%). This positive effect was reduced by the N-terminus fused protein, which indicates that the orientation of the substrate binding domain plays a critical role in the catalytic activity of the fused protein.

#### 3.4.2 Hydrophobin-fused PETase

In addition to the CBM, hydrophobins are also believed to improve the catalytic performance of PETase. They are highly surface-active proteins capable of spontaneously self-assembling at hydrophilic-hydrophobic interfaces, which are proven to be important for interface engineering (Berger and Sallada, 2019). Puspitasari et al. investigated the effects of the class I hydrophobin RolA from *Aspergillus oryzae* and HGFI from *Grifola frondose* on PETase-catalysed PET hydrolysis (Puspitasari, Tsai, and Lee, 2021a). The results showed that PET pretreatment with RolA or HGFI could all accelerate PETase hydrolysis against PET with more soluble monomers released. After pretreatment, the weight loss of hydrolysed PET increased from ∼18% to 34%; moreover, for the HGFI-modified PET fibre (crystallinity, 38.8%), the weight loss reached 34.56% after 5 days. However, no significant enhancement was detected when the simultaneous addition of hydrophobins with PETase was used for PET degradation. This finding was contrary to that obtained with class II hydrophobins (Espino-Rammer et al., 2013); therefore, this stimulation effect caused by hydrophobins should be further examined in detail. In the subsequent study, RolA was found to be able to enhance the PET hydrolysis of a PET bottle, and the highest weight loss was determined to be ∼26% after 4 days (Puspitasari, Tsai, and Lee, 2021b).

In a recent study, a zwitterionic polypeptide (5–30 kDa) consisting of alternating-charged glutamic acid (E) and lysine (K) residues was fused to the C-terminus of PETase (X. Chen, 2021). The results highlighted that the lengths of the fusion peptide play a clear role in the PET-degrading performance, and PETase-EK30 (T_m_ = 45.4°C ± 0.21°C) was found to display the highest catalytic activity (increased by 11-fold compared with the wild-type PETase) against the highly crystallized PET films (crystallinity, 45.2%). After 5 days, the concentration of products released by PETase-EK30 at 40°C against AGf-PET was further increased to 303.2 μM, 9.4-fold higher than that released by wild-type PETase. Molecular modelling revealed a strengthened structural stability and a more open substrate-binding pocket of the EKylated PETases. MD simulations indicate that the improved catalytic activity might result from the exposure of hydrophobic amino acids (W185, I208, and W159, a more open substrate-binding site), rotation of the benzene ring of Y87, and a shortened catalytic distance between the labile carbonyl carbon atom of 2PET and the hydroxy oxygen of the catalytic S160 caused by the EKylation. All these are believed to be associated with the enhanced catalytic activity for PET degradation.

In addition, the noncatalytic polysaccharide monooxygenase was shown to be capable of binding to relatively flat polysaccharide surfaces similar to CBMs (Hangasky, Detomasi, and Marletta, 2019), which might contribute to binding to the PET surface. In one study, it was reported that the lytic polysaccharide monooxygenase from *Pycnoporus coccineus* (PcAA14A) could also facilitate *Is*PETase-catalysed PET degradation (Dai, Qu, Hu, et al., 2021a). The results showed that PcAA14A alone was unable to degrade PET; however, the PET degradation products were increased to 534 ± 24.3 μM when treated with PcAA14A and *Is*PETase simultaneously, which was increased by 59.2% compared with that catalysed by *Is*PETase alone. Even in the absence of ascorbate and H_2_O_2_ (necessary for the catalytic functions of PcAA14A) or with the inactive mutant PcAA14A^H100A^, similar findings could also be obtained with 588 ± 13.5 μM, 490 ± 13.5 μM, 490 ± 26.5 μM products detected. This reveals the synergistic effects of PcAA14A and *Is*PETase in PET hydrolysis, which might be unrelated to the catalytic activity of PcAA14. After 5 days, under the optimal conditions, PET was degraded, releasing 1,097 μM products, which was increased by 27.7% compared with that produced by *Is*PETase alone. These interesting findings provide novel insights into the design of promising enzymatic machineries for PET biodegradation.

## 4 Discussion and conclusion

The plastic crisis is a global challenge that requires urgent intervention and a concerted effort that links partners across industrial, academic, financial, and government sectors. Enzymatic hydrolysis of PET, as a green and sustainable alternative, has captured great importance and interest worldwide. This strategy is considered as an environmentally friendly strategy for the recycling of post-consumer PET wastes. While progress on the PET-biodegradation has been made, there are still challenges to overcome in developing effective and scalable methods for biodegrading PET. Currently, it is the main challenge to discover and identify potential polymer-active enzymes acting on specific or different fossil-fuel-based plastics in nature or by molecular engineering of the existing enzymes. It is still imperative to further improve the thermostability of the used PETases (or PETase-like enzymes), which usually function poorly at glassy transition temperature. Though several promising PETase mutants have been reported (e.g., HotPETase, ThermoPETase and DuraPETase), molecular engineering of PETases for improved stability and PET-degrading activity simultaneously still remains a challenge but a promising strategy.

Besides, currently the mixed plastic fractions are usually difficult to recycle, however the microbial metabolism might open novel grounds with the rapid development of synthetic biology and metabolic engineering. Highly efficient microbial cell factories with modified genetic circuits would not only provide a promising strategy for PET degradation, but enable the polymer upcycling. For the upcycling strategy, the hydrolysed fragments or depolymerized monomers (e.g., as, EG, TPA) could then be used as substrates or carbon sources to produce value-added compounds such as isoprene and caprolactam. Though promising, it requires a systems-based and multidisciplinary strategy to achieve this purpose. In addition, it is necessary to determine, and cope with the factors contributing to the costs of PET-biodegradation, such as the biorefinery processes of the plastic wastes, industrialized fermentation and purification of the PET-degrading catalysts, protein engineering for improved catalytic performance and so on.

As emphasized in this review, it is believed that effective enzymes with enhanced thermostability and degradation performance combined with the rapidly developing synthetic biology and metabolic engineering tools would further streamline PET upcycling processes.
